# Group sequential designs for pragmatic clinical trials with early outcomes: methods and guidance for planning and implementation

**DOI:** 10.1186/s12874-024-02174-w

**Published:** 2024-02-16

**Authors:** Nick R. Parsons, Joydeep Basu, Nigel Stallard

**Affiliations:** Warwick Clinical Trials Unit (WCTU), Warwick Medical School, University of Warwick, CV4 7AL Coventry, UK

**Keywords:** Group sequential designs, Early outcomes, Randomized controlled trials, Planning

## Abstract

**Background:**

Group sequential designs are one of the most widely used methodologies for adaptive design in randomized clinical trials. In settings where early outcomes are available, they offer large gains in efficiency compared to a fixed design. However, such designs are underused and used predominantly in therapeutic areas where there is expertise and experience in implementation. One barrier to their greater use is the requirement to undertake simulation studies at the planning stage that require considerable knowledge, coding experience and additional costs. Based on some modest assumptions about the likely patterns of recruitment and the covariance structure of the outcomes, some simple analytic expressions are presented that negate the need to undertake simulations.

**Methods:**

A model for longitudinal outcomes with an assumed approximate multivariate normal distribution and three contrasting simple recruitment models are described, based on fixed, increasing and decreasing rates. For assumed uniform and exponential correlation models, analytic expressions for the variance of the treatment effect and the effects of the early outcomes on reducing this variance at the primary outcome time-point are presented. Expressions for the minimum and maximum values show how the correlations and timing of the early outcomes affect design efficiency.

**Results:**

Simulations showed how patterns of information accrual varied between correlation and recruitment models, and consequentially to some general guidance for planning a trial. Using a previously reported group sequential trial as an exemplar, it is shown how the analytic expressions given here could have been used as a quick and flexible planning tool, avoiding the need for extensive simulation studies based on individual participant data.

**Conclusions:**

The analytic expressions described can be routinely used at the planning stage of a putative trial, based on some modest assumptions about the likely number of outcomes and when they might occur and the expected recruitment patterns. Numerical simulations showed that these models behaved sensibly and allowed a range of design options to be explored in a way that would have been difficult and time-consuming if the previously described method of simulating individual trial participant data had been used.

**Supplementary Information:**

The online version contains supplementary material available at 10.1186/s12874-024-02174-w.

## Background

Group sequential designs (GSD) are one of the most widely used methodologies for adaptive design in randomized clinical trials [1]. In GSD researchers collect data and undertake sequential analyses with the opportunity to either reject the null hypothesis, stop the study for futility or continue recruitment at an interim look, before reaching the planned sample size [2]. Despite the self evident gains in efficiency that GSD and other adaptive designs offer due to the possibility of stopping early, the perception in much of the statistical community is that they are still underused and where they are used they are used only within niche therapeutic areas where there is expertise and experience in implementation (e.g. in pharmaceutical trials testing drugs in oncology) [1]. There has been much discussion of the reasons why this is the case and how the barriers to uptake might be overcome, in particular the lack of knowledge, experience, statistical expertise and opportunity in the clinical trials community, outside of specialist teams [3]. A recent publication showed that GSD are feasible and are likely to be considerably more efficient than fixed sample size designs for pragmatic clinical trials, an application area where adaptive designs are generally never used [4]. Pragmatic trials typically test complex interventions (e.g. surgery, exercise, cognitive behavioural therapy) in routine clinical practice and are characterised by relatively large sample sizes and long follow-up periods [5, 6]. In such settings, GSD that use data from not only the final (primary) study outcome but also from early outcomes at interim analyses to inform stopping decisions have particular attraction due in large part to the use of patient-reported outcome measures (PROMs) that show strong associations between early and final outcomes [4]. This approach is exemplified by the START:REACTS trial that used this methodology to assess a novel intervention for repair of rotator cuff tendon tears [7]. The initial design and planning of this study which was based on simulating individual trial participant data, from a multivariate distribution [8], under an assumed model for study recruitment patterns [9] in order to assess likely information accumulation during a proposed trial, is a very general and highly effective method. However, such simulations are complex and time-consuming to set-up and implement and therefore provide an additional barrier, amongst many others previously identified [3], to the widespread use of GSD, particularly for trialists and those statisticians who are not specialists in this area. If we are willing to make some modest assumptions about the distribution of the outcomes, the likely correlation structure and recruitment patterns we might expect, then we can derive relatively simple analytic expressions for information accrual during a trial. This would allow us to explore a range of options for the timing and number of interim analyses, in a routine way without the need for simulating individual participant data, and as such make the methods much more accessible to potential non-expert users. In order to do this, we propose a number of recruitment models and two contrasting correlation models for the temporal sequence of outcomes observed for individual study participants. The recruitment and correlation models together provide expressions for the variance of the treatment effect estimate and a natural means to distinguish and make explicit the contribution to the information fraction of the early and primary outcome data at an interim analysis. Previous work has discussed the timing of follow-up measurements for a single early outcome, using a simple linear model for the decay in the correlation between the final and early outcomes over time [10]. The models we develop here allow us to explore this issue in the general case of more than one early endpoint using an information adaptive group sequential approach to improve decision making at interim analyses. More generally, others have also suggested using information from prognostic baseline covariates (e.g. from baseline scores, comorbidities and patient demographics) in addition to early outcomes to inform interim decision making [11, 12]. Our focus is on stopping for treatment efficacy or futility, therefore we do not consider other adaptions that might be made to the trial design (e.g. sample size re-estimation) or more generally issues around inference and how to obtain unbiased estimates of treatment effects for group-sequential trials that stop early [13–15]. Also, given the overwhelming predominance of continuous outcomes in pragmatic trials of complex interventions, we will not discuss binary or time-to-event outcomes. Although the motivation for the work is from our own experiences with pragmatic trials, the methodological approaches described here are applicable much more widely to GSD in any application area where the issues and design characteristics we highlight are important.

We structure the paper as follows. In “Longitudinal outcomes” section, we describe a model for longitudinal outcomes with an assumed approximate multivariate normal distribution. “Recruitment and follow-up models” section develops three contrasting simple models for recruitment of participants into a clinical trial, and “Correlation models” section develops the models from “Longitudinal outcomes” and “Recruitment and follow-up models” sections for a uniform and an exponential correlation model. “Numerical examples” section provides some numerical examples to illustrate the models. The paper concludes in “Discussion” section with a discussion, including details of the availability of software for implementation of the methods described.

## Longitudinal outcomes

### A group sequential trial

Consider a two-arm randomized controlled trial where participants are randomized to either a treatment or a control arm, followed-up, assessed and the primary outcome observed at a sequence of *s* occasions at time-points $d_{1},\ldots ,d_{s}$, ordered such that $d_{s}>\dots >d_{1}$. In such a setting, the primary interest of the trial is often to estimate the effect of the treatment on the study outcome at time-point $d_{s}$, the primary or final study outcome time-point. At some time *t* during the study, the total number of participants with data at follow-up occasion *r* ($r = 1,\dots ,s$) is $N0_{r}+N1_{r}$, where $N0_{r}$ is the number in the control arm and $N1_{r}$ is the number in the treatment arm. Due to the ordering of the follow-up occasions, prior to the completion of trial follow-up, assuming data are complete, the number of participants with outcome data are structured such that $N0_{1} \ge N0_{2} \ge \dots \ge N0_{s-1} \ge N0_{s}$ and $N1_{1} \ge N1_{2} \ge \dots \ge N1_{s-1} \ge N1_{s}$. For instance, if the primary study outcome time-point is at 12 months after recruitment, with early outcomes at 3 and 6 months, then at all times prior to completion of follow-up we would expect to have more 3 month data than 6 month data, and more 3 and 6 month data than 12 month data.

If the full study sample of *N* participants is recruited in a period of time of length $\text {T}_{\text {R}}$ (the recruitment period) and the primary outcome is observed at time $d_{s}$ (after recruitment) then study follow-up is complete, and the trial ends, at time $d_{s}+\text {T}_{\text {R}}$. Importantly in this setting, there is a period of time between primary outcome data being available for analysis and the end of recruitment ($d_{s}<t<\text {T}_{\text {R}}$). During this so-called *window of opportunity*, there is the possibility of undertaking interim analyses, potentially stopping the study early for either treatment futility or efficacy. In such settings, if the interim analyses use final outcome data only, then the opportunities for stopping are likely to be extremely limited as trial recruitment will often have been completed before there is sufficient final outcome data available for informed stopping decisions to be made [4, 8]. However, if the early outcomes for trial participants (at occasions $d_{r}; r=1,\ldots ,s-1$) are correlated with their final outcomes (at $d_{s}$), then a group sequential analysis [2] which uses information from both the early and final outcomes to estimate the treatment effect at $d_{s}$ is likely to lead to considerable increases in statistical power and also to make early stopping feasible [10, 16].

A number of authors have investigated this problem [8, 10, 17] and more generally the use of group-sequential analysis for longitudinal data [18, 19]. In the most simple possible setting, for instance the double-regression method described by Engel and Walstra [17], there is a final (long-term) and a single early (short-term or concomitant) endpoint that are correlated for individuals at the two time points. The main motivation for using information from the early outcomes in addition to the final outcomes in a clinical setting is that it allows us to conduct the trial in a more efficient manner by potentially reaching a conclusive result more quickly and limiting patient exposure to ineffective or unsafe treatments, if the study ultimately provides little support for the efficacy of the intervention under test. Stallard [16], for instance, showed that using early outcome data, in the setting of a seamless phase II/III clinical trial with treatment selection, results in an increase in statistical power when data are correlated with the final outcome. A general approach in the setting of a sequential clinical trial, with a number of interim analyses, with a single long-term and potentially many short-term endpoints for a two-arm trial was first suggested by Galbraith and Marschner [10] and discussed further by Parsons et al. for a clinical trial in shoulder surgery [8], including extensive simulations for a prospective sample size calculation, and for surgical trials in general [4]. These authors rely in all cases on the independent increments argument, based on an asymptotic joint multivariate normal distribution for the sequential test statistics, for construction of valid group sequential designs for the longitudinal models used, e.g. linear mixed-effects and generalized least squares models [2, 20]. Due to the nature of the applications described, the focus here is purely on using early outcomes only to inform decision making. More generally, others have described approaches in settings where baseline (prognostic) covariates are available in addition to or in preference to early outcomes [12, 21].

### Data model

Let $y_{ijr}$ be the outcome for the $i^{th}$ of *N* participants $(i = 1,\ldots ,N)$, at follow-up occasion *r* $(r = 1,\dots ,s)$ recruited into intervention arm *j* (0 = control and 1 = treatment) of the group sequential trial. We assume hereafter independence between the trial participants and that the distribution of outcomes $(y_{ij1},\dots ,y_{ijs})$ is multivariate normal, with mean $(\mu _{j1},\dots ,\mu _{js})$ and $s \times s$ covariance matrix

1 $$\begin{aligned} \Sigma = \left( \begin{array}{cccc} \sigma ^{2}_{1} &{} \sigma _{1} \sigma _{2} \rho _{12} &{} \dots &{} \sigma _{1} \sigma _{s} \rho _{1s}\\ \sigma _{2} \sigma _{1} \rho _{21} &{} \sigma ^{2}_{2} &{} \dots &{} \sigma _{2} \sigma _{s} \rho _{2s} \\ \vdots &{} \vdots &{} \ddots &{} \vdots \\ \sigma _{s} \sigma _{1} \rho _{s1} &{} \sigma _{s} \sigma _{2} \rho _{s2} &{} \dots &{} \sigma ^{2}_{s} \end{array}\right) , \end{aligned}$$

where $\sigma _{r}$ is the standard deviation of the outcome at occasion *r* and $\rho _{rr^{\prime }}$ is the correlation between endpoints at occasions $r=1,\dots ,s$ and $r^{\prime }=1,\dots ,s$. Noting also that $\Sigma$ can be expressed as $\Sigma = \text{S}^{1/2} \text{R} \text{S}^{1/2}$, for correlation matrix $\text {R}$ and (diagonal) variance matrix $\text {S}$.

Expressing as a linear longitudinal model with correlated errors, under the assumption of multivariate normality (MVN), the vector of outcomes ${\textbf {y}}_{i}$, for participant *i*, has distribution ${\textbf {y}}_{i} \sim \text {MVN}(X_{i} \beta , \Sigma _{i})$, where $\Sigma _{i}$ is the $r \times r$ covariance matrix of ${\textbf {y}}_{i}$, for the *r* observed outcomes for participant *i*, characterised by covariance parameters $\sigma _{r}$ and $\rho _{rr^{\prime }}$. $X_{i}$ is a $r \times 2s$ design matrix and $\varvec{\beta }$ is a $2s \times 1$ vector of unknown model parameters, where for inferential purposes the most important is $\beta _{s}$ the effect of the treatment on the study outcome at time-point $d_{s}$, the primary study endpoint.

The maximum likelihood estimator for $\varvec{\beta }$, under the multivariate normal assumption, for known $\Sigma$, is the generalized least squares estimator [22]

2 $$\begin{aligned} \varvec{\beta } = \Bigg ( \sum \limits _{i=1}^N X_{i}^{\prime } \Sigma ^{-1}_{i} X_{i} \Bigg )^{-1} \Bigg ( \sum \limits _{i=1}^N X_{i}^{\prime } \Sigma ^{-1}_{i} y_{i} \Bigg ), \end{aligned}$$

with variance given by

3 $$\begin{aligned} \text {var}(\varvec{\beta }) = \Bigg ( \sum \limits _{i=1}^N X_{i}^{\prime } \Sigma ^{-1}_{i} X_{i} \Bigg )^{-1}. \end{aligned}$$

Estimates of model parameters $\varvec{\beta }$ and their variances $\text {var}(\varvec{\beta })$, and consequently information, follow naturally given $\Sigma$, which is obtained from estimates of $\varvec{\rho }$ and $\varvec{\sigma }$. The covariance parameters could, in principle, be fixed to known or expected values but are generally estimated from accumulating data as a trial progresses. For instance, Galbraith and Marschner [10] use mixed-effects models for analysis of correlated data to estimate $\varvec{\rho }$ and $\varvec{\sigma }$. In practice this can be implemented, for example, by fitting separate fixed-effects for each study outcome time $d_{r}$ with an unstructured error covariance using the function lme in R [23] package nlme. However, for practical reasons during trial planning and monitoring we prefer to use the generalized least squares model function gls in R package nlme, which unlike the mixed-effects model, provides explicit estimates of the covariance parameters [24]. Either the mixed-effects or generalized least squares formulation provides consistent and unbiased estimates of model parameters [4], under an assumed multivariate normal distribution with a general covariance structure, common follow-up times for each individual and missing outcomes that are assumed to be a consequence of the shortened follow-up duration.

### Trial planning and monitoring

The primary interest of the clinical trial is to estimate $\beta _{s}$ and its variance $\text {var}(\beta _{s})$. Easily interpretable explicit expression for $\text {var}(\beta _{s})$ do not exist for general *s*, and general covariance matrix $\Sigma$. However, expressions for $\text {var}(\beta _{s})$ can be obtained directly for the most simple cases, under the structured data assumptions of “Longitudinal outcomes” section, where there are one ($s=2$) and two ($s=3$) early outcomes [4]. For instance, for the simplest possible case $s=2$,

4 $$\begin{aligned} \text {var}(\beta _{2}) = \sigma _{2}^2\Bigg [\frac{(N0_{2}+N1_{2})(1-\rho ^{2}_{12})}{N0_{2}N1_{2}} + \frac{(N0_{1}+N1_{1})\rho ^{2}_{12}}{N0_{1}N1_{1}}\Bigg ]. \end{aligned}$$

Of particular practical importance when planning an information adaptive group sequential study is to understand how information on the treatment effect at a time *t*, $\text {I}(t)=1/\text {var}(\beta _{s}(t))$, is likely to accumulate during recruitment and follow-up. Typically pre-set expected information thresholds are used to trigger interim analyses, and to construct lower and upper stopping boundaries at the interim analyses, with stopping decisions being made based on estimates of $\beta _{s}$ and $\text {var}(\beta _{s})$ [8]. Clearly, the information at some time *t* during recruitment depends on the covariance parameters $\rho =\rho _{12},\dots ,\rho _{rr^{\prime }}$ and $\sigma _{s}$, and the number of participants ($N0_{r}$ and $N1_{r}$) with data at each follow-up occasion *r*.

In order to plan how a trial might be implemented and if, and when, interim analyses should take place, we need to understand how information is likely to accumulate as the study proceeds. To do this we need to make some *a priori* assumptions about both the expected patterns of recruitment and the correlation structure between the early and final outcomes. In the most general settings we might imagine, with complex patterns of recruitment and accrual of study data and unstructured correlations between outcomes, simulation methods may be the only way to proceed at the planning stage [8]. Such an approach is hard to implement, time-consuming and often provides little or no insight into the general principles at play and how these might guide us when we make future modifications to the design or when planning future studies. However, if we are willing to make some reasonable assumptions about the likely patterns of recruitment and the structure of the correlations then we can obtain explicit analytic expressions for $\text {var}(\beta _{s})$ much more quickly and simply, and use these as a means to plan the study.

When planning a trial we assume that a fixed number of early outcomes are available throughout the study (for instance, a primary outcome at 12 months, with two early outcomes at 3 and 6 months) and that, in principle, the timings of the early outcomes could be changed (for instance, to 4 and 8 months). Typically, the exact correlations between early and primary outcomes are unknown. However, we can speculate on the likely correlation structure as a means to understand how information might be accumulated as follow-up proceeds. Two widely used correlation models for longitudinal data are described in “Correlation models” section. At the design stage, for some arbitrarily selected time-point *t* during recruitment we will generally not know the exact number of participants recruited or the number of participants ($N0_{r}$ and $N1_{r}$) with data at each follow-up occasion *r*. We discuss simple models for predicting recruitment in “Recruitment and follow-up models” section. Given correlation and recruitment models, together with an estimate of $\sigma _{s}$ (e.g. from previously reported studies or pilot data), we can predict how $\text {var}(\beta _{s})$, and therefore information, will vary during study follow-up and use this to motivate our choice of the number and timings of early outcome assessments and interim analyses.

When monitoring a study, often more important than the information itself is the information fraction or information time $\tau (t)$ at an interim analysis at time *t*, defined by $\tau (t)=\text {I}(t)/\text {I}$, where $\text {I}(t)$ and $\text {I}$ are the information levels at time *t* and the study end, respectively [25]. Knowing the information fraction $\tau (t)$ allows us to determine lower and upper boundaries (for planned futility and efficacy stopping) and boundary crossing probabilities at an interim analysis, for some given boundary crossing probabilities under the null hypothesis, based on canonical joint distribution properties for group sequential trials [2]. Boundaries and probabilities can, for instance, be calculated using appropriate functions from the gsDesign package in R [26]. An example of how this might be implemented in practice is provided in “Numerical examples” section, using as an exemplar the START:REACTS study of sub-acromial spacer for tears affecting rotator cuff tendons [7, 27].

## Recruitment and follow-up models

Assuming the data are structured as in “A group sequential trial” section and are complete, consistent with what we would do during planning and sample size calculations in a conventional trial design based on a single primary endpoint. We can write a general expression for the number of participants providing outcome data from follow-up occasion *r* at time *t* as $N_{r}(t,d_{r})=k g_{r}(t,d_{r})$, where *k* is a constant depending on the planned sample size *N* and recruitment period $\text {T}_{\text {R}}$ only and $g_{r}(t,d_{r})$ is some function of *t* and the follow-up time point $d_{r}$, measured in the same units as *t*. For notational convenience, we define $r=0$ to be the recruitment occasion and thus $g_{0}(t,d_{0})$ is the result of the function $g_{r}(t,d_{r})$ when $r=0$, that is at the time-point when recruitment occurs at $d_{r}=0$, such that $N_{0}(t,d_{0})=k g_{0}(t,d_{0})$ is the number of participants recruited at time *t*. For $d_{r}<t\le d_{r}+\text {T}_{\text {R}}$, the number of participants is $N_{r}(t,d_{r})=k g_{r}(t,d_{r})$ and at $t<d_{r}$ prior to outcome data becoming available is $N_{r}(t,d_{r})=0$ and at $t>d_{r}+\text {T}_{\text {R}}$ when data collection has been completed for outcome *r* is $N_{r}(t,d_{r})=N$. We also note that $n_{rr^{\prime }}(t)=N_{r}(t,d_{r})/N_{r^{\prime }}(t,d_{r^{\prime }})$, the ratio of the number of study participants with outcome data from follow-up occasion $d_{r}$ to study participants with outcome data from follow-up occasion $d_{r^{\prime }}$ at time *t*, is equal to $g_{r}(t,d_{r})/g_{r^{\prime }}(t,d_{r^{\prime }})$. Introducing a weight $0<\phi <1$ that allows for unequal group sizes, gives intervention group sizes of $N0_{r}(t,d_{r})=\phi N_{r}(t,d_{r})$ and $N1_{r}(t)=(1-\phi ) N_{r}(t,d_{r})$.

### Fixed rate

**Fig. 1:**
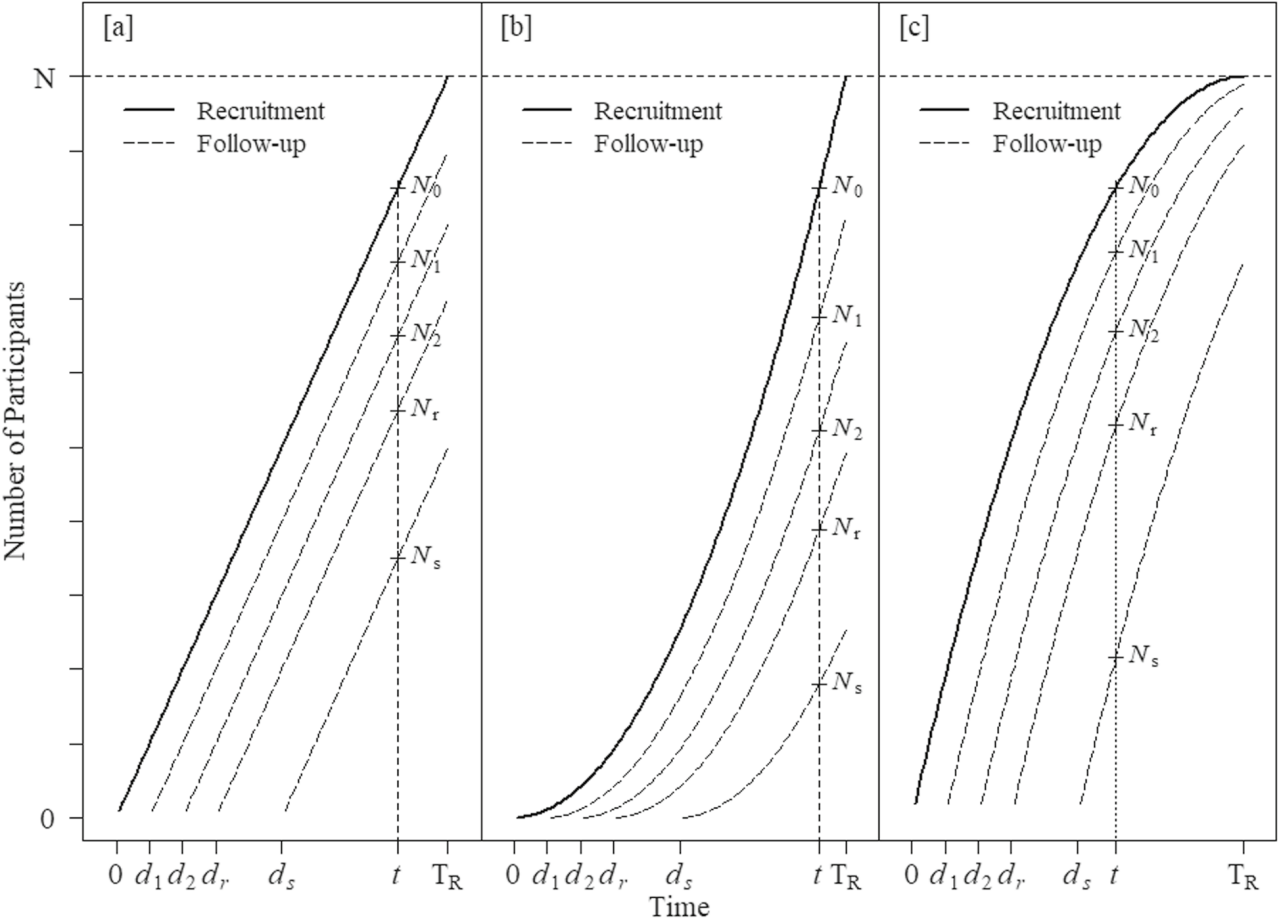
Total recruitment and follow-up accrual curves for the primary and all early follow-up endpoints at times $d_{1},d_{2},\dots ,d_{r},\dots ,d_{s}$ for (**a)** the fixed rate recruitment model, **b** the linearly increasing rate recruitment model and (**c**) the linearly decreasing rate recruitment model. With annotation showing numbers of participants recruited $N_{0}$ and with follow-up data at $N_{1},N_{2},\dots ,N_{r},\dots ,N_{s}$ at times *t*, set such that $N_{0}$ is the same for each setting

In the simplest possible situation, setting $k=N/\text {T}_{\text {R}}$ and $g_{r}(t,d_{r})=(t-d_{r})$ in the expression $N_{r}(t,d_{r})=k g_{r}(t,d_{r})$ leads to a model with a fixed rate of recruitment ($\lambda _{\text {f}}$) and follow-up data accrual, where $\lambda _{\text {f}}=N/\text {T}_{\text {R}}$ participants are recruited into the study for each of *t* study days, if $\text {T}_{\text {R}}$ is measured in days. The total number of participants recruited into the study at time *t* for the fixed model is given by $N_{0}(t)=Nt/\text {T}_{\text {R}}$; Fig. 1a shows total recruitment and follow-up data accrual curves for this model.

### Linearly increasing rate

Setting $k=N/\{\text {T}_{\text {R}}(\text {T}_{\text {R}}+1)\}$ and $g_{r}(t,d_{r})=(t-d_{r})((t-d_{r})+1)$ in the expression $N_{r}(t,d_{r})=k g_{r}(t,d_{r})$ leads to a model with an increasing rate of recruitment given by $\lambda _{\text {i}}(t)=2Nt/\{\text {T}_{\text {R}}(\text {T}_{\text {R}}+1)\}$. In this model the mean rate of recruitment across the whole recruitment period for this model is $N/\text {T}_{\text {R}}$, the same as the fixed rate parameter model $\lambda _{\text {f}}$, with the starting rate (at $t=1$) given by $\lambda _{\text {i}}(1)=2N/\text {T}_{\text {R}}(\text {T}_{\text {R}}+1)$ and the end rate (at $t=\text {T}_{\text {R}}$) by $\lambda _{\text {i}}(\text {T}_{\text {R}})=2N/(\text {T}_{\text {R}}+1)$, noting that $\lambda _{\text {i}}(1)<\lambda _{\text {f}}$ and $\lambda _{\text {i}}(\text {T}_{\text {R}})>\lambda _{\text {f}}$. The total number of participants recruited into the study at time *t* for the increasing rate model is given by $N_{0}(t)=Nt(t+1)/\{\text {T}_{\text {R}}(\text {T}_{\text {R}}+1)\}$; Fig. 1b shows total recruitment and follow-up data accrual curves for this model.

### Linearly decreasing rate

Formulating deliberately as a contrast to the model of “Linearly increasing rate” section, setting $k=N/\{\text {T}_{\text {R}}(\text {T}_{\text {R}}+1)\}$ and $g_{r}(t,d_{r})=(t-d_{r})(2\text {T}_{\text {R}}-(t-d_{r})+1)$ leads to a model with a decreasing rate of recruitment given by $\lambda _{\text {d}}(t)=2N(\text {T}_{\text {R}}-t+1)/\{\text {T}_{\text {R}}(\text {T}_{\text {R}}+1)\}$. In this model the mean rate of recruitment across the whole recruitment period for this model is also $N/\text {T}_{\text {R}}$, the same as the fixed rate parameter model $\lambda _{\text {f}}$, with the starting rate (at $t=1$) given by $\lambda _{\text {d}}(1)=2N/\{(\text {T}_{\text {R}}+1)\}$ and the end rate (at $t=\text {T}_{\text {R}}$) by $\lambda _{\text {d}}(\text {T}_{\text {R}})=2N/\{\text {T}_{\text {R}}(\text {T}_{\text {R}}+1)\}$, noting that, in a reversing of the relationship for the increasing rate model, $\lambda _{\text {d}}(1)>\lambda _{\text {f}}$ and $\lambda _{\text {d}}(\text {T}_{\text {R}})<\lambda _{\text {f}}$. The total number of participants recruited at time *t* for the decreasing rate model is given by $N_{0}(t)=Nt(2\text {T}_{\text {R}}-t+1)/\{\text {T}_{\text {R}}(\text {T}_{\text {R}}+1)\}$; Fig. 1c shows total recruitment and follow-up data accrual curves for this model.

## Correlation models

We consider two common single parameter correlation models; the uniform and exponential models [22], the latter is also known as the first-order autoregressive (AR1) model. These models offer contrasting views on the likely correlation structure between early and final outcomes. We choose to use $\alpha$ and $\gamma$ for the parameters for the uniform and exponential models, in the following descriptions, to reflect the fact that they have quite different interpretations.

### Uniform

The uniform correlation model is the natural basis for a random-effects model, which we can motivate in our setting by thinking of the covariance structure of the data as a consequence of random variation amongst (unobserved) subject-specific characteristics of participants in a clinical trial. The uniform correlation model is widely seen in trials using PROMs, where participants are asked to assess their own status or functional abilities [4]. It assumes the correlations between measurements are constant regardless of how far apart in time they are, with measurements on a unit (participant in a trial) at time-points $r=1,\dots ,s$ and $r^{\prime }=1,\dots ,s$ given by $\rho _{rr^{\prime }}=\alpha$ when $r \ne r^{\prime }$ and $\rho _{rr^{\prime }}=1$ when $r=r^{\prime }$.

#### Expression for $\text {var}(\beta _{s})$

For the uniform correlation model, assuming that the number of participants with outcome data are structured in the manner described in “Longitudinal outcomes” and “Recruitment and follow-up models” sections, the variance of the treatment effect on the study outcome at time-point *s* (the primary study endpoint) is given by

$$\begin{aligned} \text {var}(\beta ^{\text {unif}}_{s}) = \sigma _{s}^2\Bigg [\frac{N0_{1}+N1_{1}}{N0_{1}N1_{1}} + \sum \limits _{m=1}^{s-1}\frac{\det (\text {R}_{m+1})}{\det (\text {R}_{m}) }\Bigg (\frac{N0_{m+1}+N1_{m+1}}{N0_{m+1}N1_{m+1}}-\frac{N0_{m}+N1_{m}}{N0_{m}N1_{m}}\Bigg )\Bigg ], \end{aligned}$$

where $\det (\text {R}_{m})=(1-\alpha )^{m-1}(1+(m-1)\alpha )$ is the determinant of the $m \times m$ correlation matrix $\text {R}_{m}$ (see Appendix A1 for details). Therefore we can also write as follows;

5 $$\begin{aligned} \text {var}(\beta ^{\text {unif}}_{s}) ={} & {} \sigma _{s}^2\Bigg [\frac{N0_{1}+N1_{1}}{N0_{1}N1_{1}} + \nonumber \\{} & {} \sum \limits _{m=1}^{s-1}\frac{(1-\alpha )(1+m\alpha )}{(1+(m-1)\alpha ) }\Bigg (\frac{N0_{m+1}+N1_{m+1}}{N0_{m+1}N1_{m+1}}-\frac{N0_{m}+N1_{m}}{N0_{m}N1_{m}}\Bigg )\Bigg ]. \end{aligned}$$

For the simplest possible case where $s=2$, $\det (\text {R}_{1})=1$ and $\det (\text {R}_{2})=(1-\alpha )(1+\alpha )=1-\alpha ^{2}$ then, as we would expect, expressions (4) and (5) are equal (i.e. $\text {var}(\beta _{2}) = \text {var}(\beta ^{\text {unif}}_{2})$), when $\alpha =\rho _{12}$. At the extremes of $\alpha$, we note that when $\alpha =1$, then $\text {var}(\beta ^{\text {unif}}_{s}) = \sigma _{s}^2 (N0_{1}+N1_{1})/N0_{1}N1_{1}$ and we conclude that data (information) from the early outcome (at $s=1$) only are important and when $\alpha =0$, then $\text {var}(\beta ^{\text {unif}}_{s}) = \sigma _{s}^2 (N0_{s}+N1_{s})/N0_{s}N1_{s}$ and data at earlier times provide no information.

#### Recruitment models and $\text {var}(\beta _{s})$

Substituting the expressions for $N0_{r}$ and $N1_{r}$, from “Recruitment and follow-up models” section, into Eq. (5) gives the following expression for the variance of the treatment effect on the primary outcome at time *t*, where we require $d_{s}<t\le \text {T}_{\text {R}}$;

6 $$\begin{aligned} \text {var}(\beta ^{\text {unif}}_{s}(t)) ={} & {} \frac{\sigma _{s}^2}{k\phi (1-\phi )} \Bigg [\frac{1}{g_{1}(t,d_{1})} +\nonumber \\{} & {} \sum \limits _{m=1}^{s-1}\frac{(1-\alpha )(1+m\alpha )}{(1+(m-1)\alpha ) }\Bigg (\frac{1}{g_{m+1}(t,d_{m+1})}-\frac{1}{g_{m}(t,d_{m})}\Bigg )\Bigg ]. \end{aligned}$$

When the correlation is zero ($\alpha =0$) between early and primary outcomes, then $\det (\text {R}_{m+1})=\det (\text {R}_{m})=1$ for all *m* and noting that

$$\begin{aligned} \sum \limits _{m=1}^{s-1}\Bigg (\frac{1}{g_{m+1}(t,d_{m+1})}-\frac{1}{g_{m}(t,d_{m})}\Bigg )=\frac{1}{g_{s}(t,d_{s})}-\frac{1}{g_{1}(t,d_{1})}, \end{aligned}$$

then the variance when there is no correlation, $\text {var}(\beta ^{\text {0}}_{s})$, is given by

7 $$\begin{aligned} \text {var}(\beta ^{\text {0}}_{s}(t)) =\frac{\sigma _{s}^2}{k\phi (1-\phi )g_{s}(t,d_{s})}, \end{aligned}$$

where we note that $k g_{s}(t,d_{s})$ is equal to $N_{s}$ the number of study participants with primary outcome data at time *t*. We can construct a measure $\text {V}_{s}(t)$ to be the relative effect of the early outcomes, due to their correlation with the primary outcome, on reducing the variance of the primary outcome by dividing $\text {var}(\beta _{s}(t))$ by $\text {var}(\beta ^{\text {0}}_{s}(t))$. For the uniform model this is

8 $$\begin{aligned} \text {V}^{\text {unif}}_{s}(t)=n_{s1}(t) + \sum \limits _{m=1}^{s-1} \frac{(1-\alpha )(1+m\alpha )}{(1+(m-1)\alpha )}(n_{s(m+1)}(t)-n_{sm}(t)). \end{aligned}$$

Where, $n_{s1}(t)\le \text {V}^{\text {unif}}_{s}(t)\le 1$, with the lower constraint (giving the maximum possible benefit from the early outcomes) occurring when $\alpha =1$ and information on the primary outcome (at $d_{s}$) comes entirely from the first early outcome (at $d_{1}$) and the upper constraint occurring when $\alpha =0$ and there is no information from any of the early outcomes. More generally, for values of the correlation parameter between these limits, then $\text {V}^{\text {unif}}_{s}(t)$ varies as a function of both *t* and $d_{r}$ $(r = 1,\dots ,s)$, and their relative spacings.

#### Minimum and maximum of $\text {V}^{\text {unif}}_{s}(t)$

For a given value of $\alpha$, the minimum of $\text {V}^{\text {unif}}_{s}(t)$ occurs trivially for the uniform correlation model when we maximize the data available for each intermediate early outcome by moving them all towards the earliest outcome at $d_{1}$. In this setting $d_{m}\rightarrow d_{1}$ and functions $g_{m}(t,d_{m})\rightarrow g_{1}(t,d_{1})$ and consequently $n_{sm}(t)\rightarrow n_{s1}(t)$ for all $m = 2,\dots ,s-1$ and then from expression (8), noting that $n_{ss}=1$, the minimum of $\text {V}^{\text {unif}}_{s}(t)$ is given by

9 $$\begin{aligned} \min _{d_{2},\ldots ,d_{s-1}} \Big ( \text {V}^{\text {unif}}_{s}(t) \Big )=n_{s1}(t) + \frac{(1-\alpha )(1+(s-1)\alpha )}{(1+(s-2)\alpha )}(1-n_{s1}(t)). \end{aligned}$$

The maximum occurs when all the intermediate early outcomes are moved towards the final outcome at $d_{s}$. In this setting $d_{m}\rightarrow d_{s}$ and functions $g_{m}(t,d_{m})\rightarrow g_{s}(t,d_{1})$ and consequently $n_{sm}(t)\rightarrow 1$ for all $m = 2,\dots ,s-1$ and then from expression (8) the maximum of $\text {V}^{\text {unif}}_{s}(t)$ is given by

10 $$\begin{aligned} \max _{d_{2},\ldots ,d_{s-1}} \Big ( \text {V}^{\text {unif}}_{s}(t) \Big )=n_{s1}(t) + (1-\alpha ^2)(1-n_{s1}(t)). \end{aligned}$$

The terms $n_{rr^{\prime }}$ in expression (8) represent the effects of the changing sample size, with different recruitment models, and as such are independent of the correlation parameter $\alpha$. The effect of the correlation $\alpha$ on $\text {V}^{\text {unif}}_{s}(t)$ is fixed and independent of the spacing or differences between the early endpoints.

### Exponential

The exponential model, in contrast to the uniform model, assumes that the correlation between pairs of measurements on the same subject decays to zero as the time separation between them increases. This model is widely used for longitudinal outcomes [22] and evidence from our own work suggests that it is a useful working assumption for modelling the association between serial measurements of PROMs for many large pragmatic clinical trials [4]. In the exponential model the correlation between a pair of measurements on a unit (participant in a trial) at time-points *r* and $r^{\prime }$ tends towards zero as the time between measurements increases $\rho _{rr^{\prime }}=\gamma ^{|d_{r}-d_{r^{\prime }}|}$ [22]. Where the $d_{r}$ are increasing ordered times that indicates the relative times of assessment. The parameter $\gamma$ expresses the strength of association, for unit separation (i.e. where $|d_{r}-d_{r^{\prime }}|=1$), and for the applications discussed here for ease of interpretation is such that $0\le \gamma <1$.

#### Expression for $\text {var}(\beta _{s})$

For an assumed exponential model and a known, or an assumed, value of $\gamma$, then for data structured in the manner described in “Longitudinal outcomes” and “Recruitment and follow-up models” sections, $\text {var}(\beta ^{\text {exp}}_{s})$ is given after some algebraic manipulation (see Appendix A2 for details), for $s\ge 3$, by

11 $$\begin{aligned} \text {var}(\beta ^{\text {exp}}_{s}) ={} & {} \sigma _{s}^2\Bigg [\frac{(N0_{s}+N1_{s})\{1-\gamma ^{2(d_{s}-d_{s-1})}\}}{N0_{s}N1_{s}} + \nonumber \\{} & {} \sum \limits _{m=1}^{s-2} \frac{(N0_{s-m}+N1_{s-m})\{1-\gamma ^{2(d_{s-m}-d_{s-m-1})}\}\gamma ^{2(d_{s}-d_{s-m})}}{N0_{s-m}N1_{s-m}} + \nonumber \\{} & {} \frac{(N0_{1}+N1_{1})\gamma ^{2(d_{s}-d_{1})}}{N0_{1}N1_{1}}\Bigg ]. \end{aligned}$$

In this setting, without loss of generality, we can set the timings of the follow-up assessments $d_{r}$ such that in all settings $d_{1}=1$ and $d_{s}=s$ such that, for instance, if $d_{1}=1,d_{2}=3,d_{3}=7/2$ and $d_{4}=4$ then this might represent assessments at 1, 3, 3.5 and 4 years or 4, 12, 14 and 16 months, depending on whether the base unit of time is 1 year or 4 months. Although, clearly the correlation parameter $\gamma$ will generally differ depending on whether we are considering the former or latter settings. In the most general case, similar arguments can be applied if we wish to make follow-up assessments such that $d_{s}$ is not a multiple of $d_{1}$. For instance, if assessments are planned at 4, 12, 18 and 22 months, then setting $d_{1}=1,d_{2}=7/3,d_{3}=10/3$ and $d_{4}=4$ ensures that, as we would expect given the relative distances, correlations between 18 and 22 month assessments $\gamma ^{(d_{4}-d_{3})}=\gamma ^{2/3}$ are the square of those between 4 and 12 month assessments $\gamma ^{(d_{2}-d_{1})}=\gamma ^{4/3}$, for a given value of $\gamma$. If the assessments are equally spaced in our model (i.e. when $d_{r}=r$ for $r=1,\dots ,s$) and $(d_{s}-d_{s-1})=\cdots =(d_{2}-d_{1})=1$, $(d_{s}-d_{s-2})=\cdots =(d_{3}-d_{1})=2$, $\ldots$ , $(d_{s}-d_{1})=s-1$, then

12 $$\begin{aligned} \text {var}(\beta ^{\text {exp}}_{s}) ={} & {} \sigma _{s}^2\Bigg [\frac{(N0_{s}+N1_{s})(1-\gamma ^{2})}{N0_{s}N1_{s}} +\nonumber \\{} & {} (1-\gamma ^{2})\sum \limits _{m=1}^{s-2} \frac{(N0_{s-m}+N1_{s-m})\gamma ^{2m}}{N0_{s-m}N1_{s-m}} + \frac{(N0_{1}+N1_{1})\gamma ^{2(s-1)}}{N0_{1}N1_{1}}\Bigg ]. \end{aligned}$$

For the case of a single early and a final outcome, then $\text {var}(\beta ^{\text {exp}}_{2})$ is given, by dropping the middle term in the square brackets and setting $s=2$ in which case, as we might expect, $\text {var}(\beta _{2}) = \text {var}(\beta ^{\text {unif}}_{2})= \text {var}(\beta ^{\text {exp}}_{2})$, if $\alpha =\gamma$.

#### Recruitment models and $\text {var}(\beta _{s})$

Substituting the expressions for $N0_{r}$ and $N1_{r}$, from “Recruitment and follow-up models” section, into Eq. (11) gives the following expression for the variance of the treatment effect on the primary outcome at time *t*;

13 $$\begin{aligned} \text {var}(\beta ^{\text {exp}}_{s}(t)) ={} & {} \frac{\sigma _{s}^2}{k\phi (1-\phi )} \Bigg [\frac{\{1-\gamma ^{2(d_{s}-d_{s-1})}\}}{g_{s}(t,d_{s})} + \nonumber \\{} & {} \sum \limits _{m=1}^{s-2} \frac{\{1-\gamma ^{2(d_{s-m}-d_{s-m-1})}\}\gamma ^{2(d_{s}-d_{s-m})}}{g_{s-m}(t,d_{s-m})} + \frac{\gamma ^{2(d_{s}-d_{1})}}{g_{1}(t,d_{1})}\Bigg ]. \end{aligned}$$

Noting that the variance when there is no correlation ($\gamma =0$) between early and primary outcomes $\text {var}(\beta ^{\text {0}}_{s}(t))$, is given by expression (7), then the effect of the correlation, due to the early outcomes, on reducing the variance of the primary outcome for the exponential model is given by

14 $$\begin{aligned} \text {V}^{\text {exp}}_{s}(t)={} & {} 1-\gamma ^{2(d_{s}-d_{s-1})} + \nonumber \\{} & {} \sum \limits _{m=1}^{s-2}n_{s(s-m)}(t) (1-\gamma ^{2(d_{s-m}-d_{s-m-1})})\gamma ^{2(d_{s}-d_{s-m})} + n_{s1}(t)\gamma ^{2(d_{s}-d_{1})}. \end{aligned}$$

Where $n_{s1}(t)\le \text {V}^{\text {exp}}_{s}(t)\le 1$, with the lower constraint occurring when $\gamma =1$ and information on the primary outcome (at $d_{s}$) comes entirely from the first early outcome (at $d_{1}$) and the upper constraint occurring when $\gamma =0$ and there is no information from any of the early outcomes. More generally, for values of the correlation parameter between these limits, then $\text {V}^{\text {exp}}_{s}(t)$ varies as a function of both *t* and $d_{r}$ $(r = 1,\dots ,s)$, and their relative spacings.

#### Minimum and maximum of $\text {V}^{\text {exp}}_{s}(t)$

The maximum of $\text {V}^{\text {exp}}_{s}(t)$, for known $\gamma$, occurs when $d_{s-1} \rightarrow d_{1}$ and as a consequence all the intermediate terms move also towards $d_{1}$; i.e. $d_{s-1} \rightarrow d_{s-2} \rightarrow \dots \rightarrow d_{2} \rightarrow d_{1}$, is given by

15 $$\begin{aligned} \max _{d_{2},\ldots ,d_{s-1}} \Big ( \text {V}^{\text {exp}}_{s}(t) \Big )=n_{s1}(t) + (1-\gamma ^{2(d_{s}-d_{1})})(1-n_{s1}(t)). \end{aligned}$$

In contrast to the uniform correlation model, there is no simple expression for the minimum of $\text {V}^{\text {exp}}_{s}(t)$. As we move intermediate outcomes towards the earliest outcome $d_{m}\rightarrow d_{1}$ then functions $g_{m}(t,d_{m})\rightarrow g_{1}(t,d_{1})$ for all $m = 2,\dots ,s-1$ and we have more data available which, all other things being equal, will minimise $\text {V}^{\text {exp}}_{s}(t)$. However, when we increase the amount of data available by moving intermediate outcomes towards the earliest outcome we also increase the distances $d_{s}-d_{s-m}$ which, from expression (14), clearly acts to increase $\text {V}^{\text {exp}}_{s}(t)$ by making terms $\gamma ^{2(d_{s}-d_{s-m})} \rightarrow 0$.

The settings of $d_{2},\ldots ,d_{s-1}$ that minimise $\text {V}^{\text {exp}}_{s}(t)$, will vary with the correlation parameter $\gamma$ and *s*. In general, minimums of $\text {V}^{\text {exp}}_{s}(t)$ can be obtained numerically using linearly constrained optimization methods; e.g. using function constrOptim in R, with gradients set to be the derivatives $\partial \text {V}^{\text {exp}}_{s} / \partial d_{m}$, which are relatively simple to calculate (see Appendix A3 for details) [23].

### Information

The information fraction, when the correlation between early outcomes and the final outcome is zero ($\tau \text {0}$) at time *t*, is given by the information at time *t* divided by the information at the study end which is $\tau \text {0}(t)=\text {var}(\beta ^{\text {0}}_{s}(t=d_{s}+T_{R}))/\text {var}(\beta ^{\text {0}}_{s}(t))$ [25]; noting that at the study end $t=d_{s}+T_{R}$. From expression (7), this can be written more simply as $\tau \text {0}(t)=N_{s}(t,d_{s})/N$, the proportion of participants with final outcome data at time *t*, or alternatively as $\tau \text {0}(t)=g_{s}(t,d_{s})/g_{s}(d_{s}+\text {T}_{\text {R}},d_{s})$.

From previously, the relative effect of the early outcomes, due to their correlation with the primary outcome, on reducing the variance of the primary outcome is $\text {V}_{s}(t)=\text {var}(\beta _{s}(t))/\text {var}(\beta ^{\text {0}}_{s}(t))$. Therefore, the information fraction $\tau$ at time *t*, for the full longitudinal model including the contribution of the early outcomes, is given by

16 $$\begin{aligned} \tau (t)=\tau \text {0}(t)/\text {V}_{s}(t). \end{aligned}$$

This allows us to make explicit the distinction between information that comes directly from observation of the final outcome ($\tau \text {0}$) and information that comes from the early outcomes ($\text {V}_{s}$) at time *t*. It also makes clear that $1/\text {V}_{s}(t)$ is the proportionate increase in the information fraction $\tau$ at time *t* due to the early outcomes. For instance, if $\text {V}_{s}(t)=0.8$, then we have 1.25 times as much information at time *t* than we would have had if the early outcomes were uncorrelated with the primary outcome.

## Numerical examples

### Uniform correlation model

To understand the properties of the uniform correlation model in the setting described, we calculate $\text {V}^{\text {unif}}_{s}$ for typical values of $s=2,3,4,5,6$ for the recruitment models of “Recruitment and follow-up models” section. Without loss of generality, we set $d_{s}=2$ and $d_{1}=1$ and arbitrarily set the recruitment period $\text {T}_{\text {R}}$ to be a fixed multiple of $d_{s}$ such that the information fraction $\tau \text {0}(t_{w})$ when $\alpha =0$ (i.e. the proportion of participants with final outcome data), at three equally spaced interim analyses ($w=1,2,3$), are $\tau \text {0}(t_{1})=0.15$, $\tau \text {0}(t_{2})=0.30$ and $\tau \text {0}(t_{3})=0.45$, which we nominally refer to hereafter as *early*, *mid* and *late*. The actually timings of the interim analyses, relative to $\text {T}_{\text {R}}$, will depend on the selected recruitment model; see Appendix A4 for details. We do this to allow us to make simple comparisons between the recruitment models and values of *s* at each the interim analyses.

Plots showing the difference in minimum and maximum values and the empirical distribution of $\text {V}^{\text {unif}}_{s}$ for equal group sizes ($\phi =0.5$), with varying $d_{2},\dots ,d_{s-1}$, for correlations in the range $0\le \alpha < 1$ and the decreasing, fixed and increasing rate recruitment models are available in the supplementary files (see Additional file 1, Figs. S1 to S9). The pattern of differences between recruitment models and interim analyses are consistent across values of $\alpha$, with values for $\text {V}^{\text {unif}}_{s}$ decreasing monotonically with increasing $\alpha$; the larger the correlation, the greater the information available from the early outcomes. Picking a typical value of $\alpha =0.5$ for illustration purposes, Table 1 shows the effects of *s*, recruitment model and timing of interim analysis on $\text {V}^{\text {unif}}_{s}$ and minimum and maximum values of $\text {V}^{\text {unif}}_{s}$ and also, as a means of comparison the equal spacing model where $d_{r}=1+(r-1)/(s-1)$ for all $r=1,\ldots ,s$, which we denote by $\tilde{\text {V}}^{\text {unif}}_{s}$.

Table 1 and expressions (8), (9) and (10) show that variation in $\text {V}^{\text {unif}}_{s}$, for some fixed *s*, is due solely to differences in $n_{s1}$, the ratio of the number of study participants with final outcome data to study participants with first early outcome data at time *t*. The value of $n_{s1}$ depends on both the timing of the interim analysis and the recruitment model. As $n_{s1}\rightarrow 1$, when all participants have final outcome data, then also $\text {V}^{\text {unif}}_{s}\rightarrow 1$ and there is no additional information available from the early outcomes. This is apparent in the trend for larger values in $\text {V}^{\text {unif}}_{s}$, for all settings, as we move from early to mid to late interim analyses and $n_{s1}$ increases in value. As we increase the number of early outcomes for fixed $\alpha$, with increasing *s*, then the term $\text {D}=(1-\alpha )(1+(s-1)\alpha )/(1+(s-2)\alpha )$ in expression (8) decreases in value towards a minimum of $(1-\alpha )$ as $s\rightarrow \infty$. Values of *D* in Table 1, for $\alpha =0.5$, decrease with increasing *s* rapidly initially (e.g. from $s=2$ to $s=3$), but slower later (e.g. from $s=5$ to $s=6$). This suggests that there is little to be gained in increasing information fraction by increasing *s* much beyond the values we use here.

**Table 1:** Minimum and maximum values of $\text {V}^{\text {unif}}_{s}$ and values for the equal spacing model $\tilde{\text {V}}^{\text {unif}}_{s}$, for $\alpha =0.5$ for early ($t_{1}$), mid ($t_{2}$) and late ($t_{3}$) interim analyses, where $\tau \text {0}_{1}=0.15$, $\tau \text {0}_{2}=0.30$ and $\tau \text {0}_{3}=0.45$ respectively, for *s* from two to six for the fixed, increasing and decreasing rate recruitment models. Also shown are values of $\text {D}=(1-\alpha )(1+(s-1)\alpha )/(1+(s-2)\alpha )$ and $n_{s1}$ for each setting

		Early $(t_{1})$			Mid $(t_{2})$			Late $(t_{3})$		
s	D	$n_{s1}$	$\tilde{\text {V}}^{\text {unif}}_{s}$	$\text {V}^{\text {unif}}_{s}$	$n_{s1}$	$\tilde{\text {V}}^{\text {unif}}_{s}$	$\text {V}^{\text {unif}}_{s}$	$n_{s1}$	$\tilde{\text {V}}^{\text {unif}}_{s}$	$\text {V}^{\text {unif}}_{s}$
				min - max			min - max			min - max
[a] Fixed										
2	0.75	0.55	-	0.89-0.89	0.71	-	0.93-0.93	0.78	-	0.95-0.95
3	0.67	0.55	0.86	0.85-0.89	0.71	0.91	0.90-0.93	0.78	0.94	0.93-0.95
4	0.62	0.55	0.85	0.83-0.89	0.71	0.90	0.89-0.93	0.78	0.93	0.92-0.95
5	0.60	0.55	0.84	0.82-0.89	0.71	0.90	0.88-0.93	0.78	0.92	0.91-0.95
6	0.58	0.55	0.83	0.81-0.89	0.71	0.89	0.88-0.93	0.78	0.92	0.91-0.95
[b] Increasing										
2	0.75	0.59	-	0.90-0.90	0.68	-	0.92-0.92	0.72	-	0.93-0.93
3	0.67	0.59	0.88	0.86-0.90	0.68	0.90	0.89-0.92	0.72	0.92	0.91-0.93
4	0.62	0.59	0.86	0.84-0.90	0.68	0.89	0.88-0.92	0.72	0.91	0.90-0.93
5	0.60	0.59	0.85	0.83-0.90	0.68	0.89	0.87-0.92	0.72	0.90	0.89-0.93
6	0.58	0.59	0.85	0.83-0.90	0.68	0.88	0.87-0.92	0.72	0.90	0.88-0.93
[c] Decreasing										
2	0.75	0.42	-	0.86-0.86	0.62	-	0.91-0.91	0.74	-	0.93-0.93
3	0.67	0.42	0.82	0.81-0.86	0.62	0.88	0.87-0.91	0.74	0.92	0.91-0.93
4	0.62	0.42	0.80	0.78-0.86	0.62	0.87	0.86-0.91	0.74	0.91	0.90-0.93
5	0.60	0.42	0.79	0.77-0.86	0.62	0.86	0.85-0.91	0.74	0.91	0.89-0.93
6	0.58	0.42	0.78	0.76-0.86	0.62	0.86	0.84-0.91	0.74	0.90	0.89-0.93

### Exponential correlation model

The correlation between the primary outcome at $d_{s}$ and the first outcome at $d_{1}$ is given by $\gamma ^{d_{s}-d_{1}}$, and the parameter $\gamma$ can be set such that the known or expected correlation between $d_{1}$ and $d_{s}$ is $\rho _{1s}$. Therefore, as a means to produce consistency between uniform and exponential correlation models we set $\gamma =\alpha ^{1/(d_{s}-d_{1})}$, where $\alpha$ is the reference correlation from the uniform model. As $d_{s}=2$ for all *s*, then $\gamma =\alpha$. Using these parametrisations for $d_{r}$ and $\gamma$, we note that by replacing $\gamma$ in expression (15) by $\alpha$, $d_{s}=2$ and $d_{1}=1$, results in $\max (\text {V}^{\text {unif}}_{s})=\max (\text {V}^{\text {exp}}_{s})$.

Plots showing the difference in minimum and maximum values and the empirical distribution of $\text {V}^{\text {exp}}_{s}$ for equal group sizes ($\phi =0.5$), with varying $d_{2},\dots ,d_{s-1}$, for correlations in the range $0\le \gamma < 1$ and the decreasing, fixed and increasing rate recruitment models are available in the supplementary files (see Additional file 1, Figs. S10 to S18). The pattern of differences between recruitment models and interim analyses are consistent across values of $\gamma$, with values for $\text {V}^{\text {exp}}_{s}$ decreasing monotonically with increasing $\gamma$; the larger the correlation, the greater the information available from the early outcomes. Therefore, as for the uniform model, we pick a typical value of $\gamma =0.5$ to illustrate in Table 2 the effects of *s*, recruitment model and timing of interim analysis on $\text {V}^{\text {exp}}_{s}$.

Table 2 shows minimum and maximum values of $\text {V}^{\text {exp}}_{s}$ and also the equal spacing model. The values of $\tilde{\text {V}}^{\text {exp}}_{s}$ in Table 2 are always smaller than values of $\tilde{\text {V}}^{\text {unif}}_{s}$ in Table 1. This is due to the settings we adopt here to force $\max (\text {V}^{\text {unif}}_{s})=\max (\text {V}^{\text {exp}}_{s})$, that make the correlations in the exponential model stronger. For instance, for the model where $s=3$, the correlation between the early outcome at $d_{2}$ and the final outcome at $d_{3}$ is given by $\alpha$ in the uniform model and $\sqrt{\alpha }$ (i.e. $\alpha ^{d_{3}-d_{2}}=\alpha ^{2-1.5}$) in the exponential model. The observed decreases in $\text {V}^{\text {exp}}_{s}$ as we move from $s=2$ to $s=6$, across all settings, are of a similar magnitude to those observed for the uniform model, suggesting some gains in information with increasing numbers of early outcomes, but with those gains diminishing as *s* increases. Of particular note for the exponential model is that, for the selected correlation $\gamma =0.5$, $\tilde{\text {V}}^{\text {exp}}_{s}$ is quite close to $\min (\text {V}^{\text {exp}}_{s})$, across recruitment models and interim analyses. This suggests that for a moderate correlation, having equally spaced outcomes is very close to the best possible design setting. As correlations become larger and $\gamma$ approaches one, then clearly the spacing of the outcomes becomes unimportant. Conversely, as the correlation becomes smaller then the spacing of the outcomes has a larger relative impact on $\text {V}^{\text {exp}}_{s}$, for the recruitment models we explore here, and suggests that moving the early outcomes to be nearer to the final outcome provides more information.

**Table 2:** Minimum and maximum values of $\text {V}^{\text {exp}}_{s}$ and values for the equal spacing model $\tilde{\text {V}}^{\text {exp}}_{s}$, for $\gamma =0.5$ for early ($t_{1}$), mid ($t_{2}$) and late ($t_{3}$) interim analyses, where $\tau \text {0}_{1}=0.15$, $\tau \text {0}_{2}=0.30$ and $\tau \text {0}_{3}=0.45$ respectively, for *s* from two to six for the fixed, increasing and decreasing rate recruitment models. Also shown are values of $n_{s1}$ for each setting

	Early $(t_{1})$			Mid $(t_{2})$			Late $(t_{3})$		
s	$n_{s1}$	$\tilde{\text {V}}^{\text {exp}}_{s}$	$\text {V}^{\text {exp}}_{s}$	$n_{s1}$	$\tilde{\text {V}}^{\text {exp}}_{s}$	$\text {V}^{\text {exp}}_{s}$	$n_{s1}$	$\tilde{\text {V}}^{\text {exp}}_{s}$	$\text {V}^{\text {exp}}_{s}$
			min - max			min - max			min - max
[a] Fixed									
2	0.55	-	0.89-0.89	0.71	-	0.93-0.93	0.78	-	0.95-0.95
3	0.55	0.81	0.80-0.89	0.71	0.88	0.88-0.93	0.78	0.92	0.91-0.95
4	0.55	0.78	0.78-0.89	0.71	0.87	0.86-0.93	0.78	0.90	0.90-0.95
5	0.55	0.77	0.76-0.89	0.71	0.86	0.85-0.93	0.78	0.90	0.90-0.95
6	0.55	0.76	0.75-0.89	0.71	0.85	0.85-0.93	0.78	0.89	0.89-0.95
[b] Increasing									
2	0.59	-	0.90-0.90	0.68	-	0.92-0.92	0.72	-	0.93-0.93
3	0.59	0.83	0.83-0.90	0.68	0.87	0.87-0.92	0.72	0.89	0.89-0.93
4	0.59	0.81	0.80-0.90	0.68	0.85	0.85-0.92	0.72	0.88	0.87-0.93
5	0.59	0.80	0.79-0.90	0.68	0.84	0.84-0.92	0.72	0.87	0.87-0.93
6	0.59	0.79	0.79-0.90	0.68	0.84	0.84-0.92	0.72	0.86	0.86-0.93
[c] Decreasing									
2	0.42	-	0.86-0.86	0.62	-	0.91-0.91	0.74	-	0.93-0.93
3	0.42	0.75	0.73-0.86	0.62	0.84	0.84-0.91	0.74	0.89	0.89-0.93
4	0.42	0.71	0.69-0.86	0.62	0.82	0.82-0.91	0.74	0.88	0.88-0.93
5	0.42	0.69	0.68-0.86	0.62	0.81	0.80-0.91	0.74	0.87	0.87-0.93
6	0.42	0.67	0.67-0.86	0.62	0.80	0.80-0.91	0.74	0.87	0.86-0.93

### START:REACTS clinical trial

The START:REACTS study was a double-blind, group-sequential, randomised controlled trial for rotator cuff tendon (shoulder) tears comparing arthroscopic debridement of the subacromial space with biceps tenotomy (control group) with the same procedure but including insertion of a sub-acromial spacer balloon (treatment group) [7, 27]. At the planning stage, individual participant data were simulated, for 10000 trials, and the models described in “Data model” section were fitted in order to estimate treatment effects, test statistics and information for each simulated trial; details of simulations and the how they were implemented are reported in detail by Parsons et al. [8]. The results of the simulations for START:REACTS showed that for 90% power, a minimum of $N=188$ participants were required, with the expected number of participants providing outcome data and trial information at the interim analyses shown in Table 3[a]. The expected information at the study end was given by $\text {I}=N/(4\sigma _{s}^2)=188/(4\times 144)=0.326$. Table 3[b] shows the observed numbers of participants providing data and the information and the estimated test statistic ($Z=\beta _{s}/\text {sd}(\beta _{s})$) at the first interim analysis when the study was stopped for futility.

**Table 3:** START:REACTS study planning and observed trial data. Numbers of participants providing outcome data at 3, 6 and 12 months, information ($\text {I}=1/\text {var}(\beta _{s})$) and test statistic ($Z=\beta _{s}/\text {sd}(\beta _{s})$) boundaries in [a] the expected (planned) study design based on extensive simulations and [b] observed in the trial itself. Note that the trial was stopped at the first interim analysis for futility, as the test statistic fell below the lower boundary

[a] Expected					[b] Observed		
Outcome		Interim		End	Interim		End
		1	2		1	2	
12 months	$N_{3}$	50	70	188	47	-	-
6 months	$N_{2}$	90	110	188	86	-	-
3 months	$N_{1}$	120	140	188	112	-	-
Information	*I*	0.102	0.139	0.326	0.110	-	-
Information fraction	$\tau$	0.311	0.426	1.000	-	-	-
Test statistic	*Z*	-	-	-	-0.881	-	-
Boundary	lower	-0.706	0.581	1.909	-	-	-
	upper	$\infty$	3.090	1.909	-	-	-

The observed correlations between outcomes were larger than expected at the first interim analysis when the trial was stopped; $\alpha \approx 0.75$, not $\alpha =0.5$ as planned. Although the observed numbers of participants providing outcome data were reasonably similar to the expected numbers, this was more by chance than by design, as the number of recruiting sites used for the trial was actually 24, not the planned 15, and the pattern of site initiations was quite different from the plan.

The expectation in the original study design was that the interim analyses should occur when approximately 25% and 35% of the trial participants had final outcome data; that is when $\tau \text {0}(t_{1})=0.25$ and $\tau \text {0}(t_{2})=0.35$. For the uniform correlation model, setting $d_{r}$ for $r=1,2,3$ to reflect the spacing of the outcomes at 3, 6 and 12 months (e.g. we can set $d_{1}=1$, $d_{2}=2$ and $d_{3}=4$) and $T_{R}=2d_{3}$ (i.e. the recruitment period is 24 months and the final outcome is at 12 months) allows us to calculate $\text {V}^{\text {unif}}_{3}(t)$ at $t=t_{1}$ and $t=t_{2}$. From expression (8), in “[Sec Sec13]” section and setting $\alpha =0.5$, these are as follows $\text {V}^{\text {unif}}_{3}(t_{1})=0.808$ and $\text {V}^{\text {unif}}_{3}(t_{2})=0.836$ for $t_{1}=6$ (18 months) and $t_{2}=6.8$ (20.4 months), the times when $\tau \text {0}(t_{1})=0.25$ and $\tau \text {0}(t_{2})=0.35$, for the example formulations for $d_{r}$. From expression (16), in “Information” section, the information fractions at these interim analyses are $\tau (t_{1})=0.309$ and $\tau (t_{2})=0.419$. The information fractions allow us to calculate bounds and probabilities (power), using for instance functions gsBound and gsProbability from the R package gsDesign [26], for selected values of the overall trial sample size *N*. Table 4[a] shows the expected numbers of participants providing data and the information and test statistic boundaries at the first and second interim analyses for the fixed recruitment model for $N=188$. Power for the fixed recruitment rate made is 90.6% for a treatment difference of 6 and $\sigma _{3}=12$, the same as the actual START:REACTS trial.

We can repeat the above calculation quite simply for the decreasing rate recruitment model, recruiting over the same length of time at the same three time-points (3, 6 and 12 months) and, for the same choice of covariance parameters, we get $\text {V}^{\text {unif}}_{3}(t_{1})=0.786$ and $\text {V}^{\text {unif}}_{3}(t_{2})=0.820$ for $t_{1}=5.13$ (15.4 months) and $t_{2}=5.64$ (16.9 months) with information fractions at these interim analyses of $\tau (t_{1})=0.318$ and $\tau (t_{2})=0.427$. Table 4[b] shows the expected numbers of participants providing data and the information and test statistic boundaries at the first and second interim analyses for the decreasing recruitment model for $N=188$. Power for the decreasing recruitment rate made is 90.7%.

**Table 4:** Numbers of participants providing outcome data at 3, 6 and 12 months, information ($\text {I}=1/\text {var}(\beta _{s})$) and test statistic ($Z=\beta _{s}/\text {sd}(\beta _{s})$) boundaries for putative START:REACTS trial designs where the sample size is $N=188$ for [a] an expected fixed rate and [b] a decreasing rate of recruitment

[a] Fixed rate					[b] Decreasing rate		
Outcome		Interim		End	Interim		End
		1	2		1	2	
12 months	$N_{3}$	47.0	65.8	188	47.0	65.8	188
6 months	$N_{2}$	94.0	112.8	188	113.5	127.0	188
3 months	$N_{1}$	117.5	136.3	188	138.9	149.8	188
Information	*I*	0.101	0.137	0.326	0.104	0.139	0.326
Information fraction	$\tau$	0.309	0.419	1.000	0.318	0.427	1.000
Boundary	lower	-0.706	0.581	1.907	-0.706	0.581	1.910
	upper	$\infty$	3.090	1.907	$\infty$	3.090	1.910

**Fig. 2:**
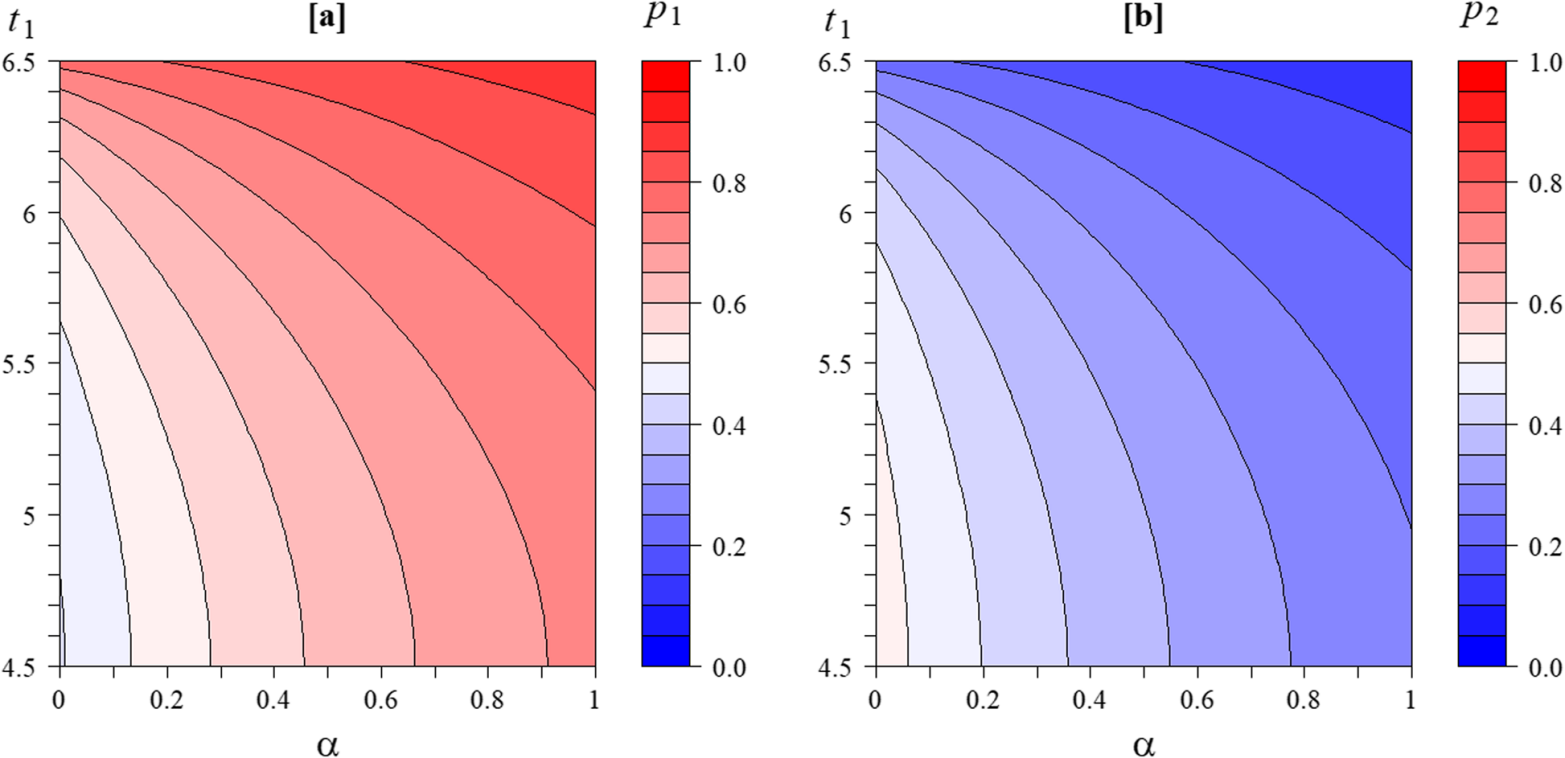
Contour plots showing futility stopping probabilities for a treatment difference of -4 (in favour of the control group) for the fixed rate recruitment and the uniform correlation model at (**a**) the first interim ($p_{1}$) and (**b**) the second interim analysis ($p_{2}$) as functions the correlation $\alpha$ and the timing of the first interim analysis ($t_{1}$; in 3 monthly base unit, such that for instance $t_{1}=5$ corresponds to 15 months)

The final trial reported a strong effect in favour of the control group -4$\cdot$2 (95% CI -8$\cdot$2 to -0$\cdot$26), rather than the expected effect in favour of the treatment group [7]. If a result of this magnitude in favour of the control group had been anticipated, then we would have had the probabilities $p_{1}$ and $p_{2}$ of stopping for futility at the interim analyses shown in Fig. 2a and b, as functions of $\alpha$ and the first analysis time-point $t_{1}$. At the extreme of the latter values shown at $t_{1}=4.5$ (13.5 months) there would have been very little outcome data for the fixed rate model ($N_{3}=11.8$) and a consequent modest value of $p_{1}=0.446$, with $p_{2}=0.525$ ($N_{3}=65.8$), assuming no correlation $\alpha =0$. Increasing the correlation to $\alpha =0.8$ would provide a considerably larger first interim analysis futility stopping probabilities $p_{1}=0.581$. A later first interim analysis at 19.5 months ($t_{1}=6.5$) would provide much more data ($N_{3}=58.8$) and a larger probability of stopping $p_{1}=0.716$ for $\alpha =0$ ($p_{1}=0.820$ at $\alpha =0.8$).

## Discussion

The numerical examples of “Numerical examples” section show how patterns of information accrual vary between correlation and recruitment models and provide some general guidance for planning a group sequential trial with early outcome data. Given equal numbers of participants providing early and final outcome data, the stronger the correlation between early and final outcomes the greater the gain in information (reduction in variance) at some interim time-point *t*. Expressions (8) and (14) show $\text {V}_{s}(t)$, the relative effect of the early outcomes on reducing the variance of the primary outcome, for the uniform and exponential models to be monotonically increasing in the correlation parameters ($0\le \alpha <1$ and $0\le \gamma <1$) under our model constraints. Therefore, if a number of suitable outcome measures are available then one should choose the one with the strongest correlations between serial time-points, assuming that the measures are equally responsive to change and have similar variances. Making recommendations for the number and, particularly, the timing of early outcomes is more complex. The simulations (Tables 1 and 2) show that there was generally little change in $\text {V}_{s}$ for $s>5$, suggesting that for the models tested there was little to be gained by having more than four early outcome time-points. For the exponential model, equally spaced early outcomes, for known $\gamma$, proved to be a sensible, and often almost optimal, choice to give the greatest information gain (i.e. values of $\tilde{\text {V}}^{\text {exp}}_{s}$ are close to $\min (\text {V}^{\text {exp}}_{s})$). For smaller values of $\gamma$ there was some value for the exponential model in moving the early outcomes to be nearer to the final outcome. However, one might argue that such an approach is not sensible for such small values of $\gamma$ as little would be gained in practice by waiting to collect early outcomes at such a late time-point. For the time invariant uniform correlation model, maximum information gains are made (trivially) when the early outcomes occur as early as possible, simply because more data will be available as more trial participants will been followed-up. Clearly, it is not sensible to assume that the uniform model must apply for any spacing between outcome time-points. Therefore, it is worth emphasising that the results presented in Tables 1 and 2 are strongly dependent on the model assumptions, in the sense that we have assumed that changing the timings of the early outcomes is possible for some fixed value of the correlation parameter. For instance, if there was known to be an equal correlation of magnitude $\alpha$ between four equally-spaced outcome time-points, then would this correlation model still be appropriate if the first two outcomes and the last two outcomes were moved to be almost coincident? It seems highly unlikely. This being the case, we would caution against using numerical experiments (such as those of “Uniform correlation model” and “Exponential correlation model” sections) solely as a means to make decisions on the spacing of outcome time-points. However, we do believe that within reasonable limits it would be profitable to explore the likely information gains that alternate spacing models may offer. Although, such decisions may be dependent on the specific application area and other trial constraints such as when participant follow-up would routinely be available. One aspect of the numerical simulation studies that is clear is that the decreasing recruitment rate model is preferable, in terms of information gain, to either the increasing or fixed rate models. This is due to a greater proportion of the study participants being available to provide early outcome data for the decreasing rate model. In many instances, there is little one could do about the likely pattern of recruitment into a trial, but in settings where this was possible then clearly it would be advantageous to plan to recruit a large proportion of the target sample size into the trial as early as possible.

The STARTS:REACTS trial provided a real example of a study using an information adaptive approach for a group sequential trial with early outcome data [7]. The study was originally planned based on a large study that simulated individual participant data [8]. What the results of “START:REACTS clinical trial” section show is that the trial could have been planned, with little effort and without the need for time-consuming coding and simulation, based on the models described here. If that had been done, then, as Tables 3 and 4 show, the final design would have been almost exactly equivalent to that used in the original study, at considerably less effort and cost. Additionally, a range of other options (e.g. changing the number and timing of interim analysis) could have been explored with none of the considerable extra work and effort that would have been required if we were simulating outcomes for individual participants. An advantage of using the approaches to design outlined here, rather than simulation models, is that the procedures are absolutely specified and explicit, and therefore easily checked and replicated by others. Whereas, in a simulation study much relies on the availability of the code and the readability and competence of the coding and also the assumptions made by those developing the code, that are often not explicitly stated. For these, and many other reasons, we would strongly recommend that those wishing to use the group sequential designs described here use the one of the selection of models in “Correlation models” section. Clearly, if one believes that the study setting is of a completely different type, or does not approximate to one of the settings described here then simulation may be the only option to determining appropriate trial sample sizes.

This study presents certain limitations. The choices of fixed, decreasing and increasing recruitment rate models (“Recruitment and follow-up models” section) was essentially arbitrary and used mainly as a means of showing a range of contrasting options. The fixed rate model might represent for instance a situation where participants were identified and recruited into a study at a fixed rate across one or more recruitment centres. Whereas the increasing rate model might arise naturally if the number of centres was likely to increase during recruitment and each centre recruited at the same fixed rate, resulting in an overall recruitment rate that followed the profile seen in Fig. 1b. The decreasing rate model Fig. (1c) provides the type of recruitment profile that might be observed where there is an existing pool of participants who are available to enter a trial quickly, resulting in a rapid rise that is followed by a slowing rate of accrual after the pool is exhausted and we have to rely solely on a new (incident) cases of a condition being identified. All of these scenarios are amongst the many we have observed within our own trials experience. Although we fully accept that the settings we present may not cover every possible option that trialists using these methods may wish to consider. However, we believe it would be relatively easy to suggest and implement a range of other sensible models, provided they followed the general structures and properties we outline in “Recruitment and follow-up models” section. Similarly, although clearly it would be possible to consider more complex correlation models than those described in “Correlation models” section, we chose the uniform and exponential correlation models mainly because they are very widely used for longitudinal outcomes, are often good approximations to observed data and also because they lead to simple analytic expressions for $\text {var}(\beta _{s})$, and as such allow us to illustrate some key ideas about the methodological approach described here [4, 22]. In practice, if we wished to assume that outcomes followed an exponential correlation model based on limited data, then we could reason as follows. For instance, consider a study that is being planned with four outcomes at 3, 6, 12 an 18 months, with the latter as the final (primary) outcome, and the others as early outcomes. Data from another study suggests that the correlation between outcomes at 3 and 12 months is approximately $\rho _{3m,12m}=0.5$, and therefore by noting that $\gamma =\rho _{rr^{\prime }}^{1/{|d_{r}-d_{r^{\prime }}|}}$, we can write $\gamma =0.5^{1/{3}}$, by setting $d_{1}=1$, $d_{2}=2$, $d_{3}=4$ and $d_{4}=6$ to model the outcome spacings. In this model, $\rho _{3m,6m}=0.79$, $\rho _{3m,12m}=0.50$, $\rho _{3m,18m}=0.31$, $\rho _{6m,12m}=0.63$, $\rho _{6m,18m}=0.40$ and $\rho _{12m,18m}=0.63$. The simple expressions for $\text {var}(\beta _{s})$ for the exponential and uniform correlation models are due to the fact that general expressions are available for $\text {R}^{-1}_{s}$; see Appendix A1 and A2. Therefore, if similar general expressions were available for alternate correlation models, then in principle we believe it would be possible to provide analytic expressions for such models.

Currently, those who wish to exploit the methodology reported here will need to implement the results themselves. However, work is ongoing to develop a package of R functions [23] to implement the models in a form that will make it easy for the user to explore all the design options described in a simple and interactive manner.

## Conclusions

We have developed models for information accrual during recruitment into a group sequential clinical trial using early outcomes to augment the information available from the trial primary outcome measures as a means to make decisions about whether to stop prior to the completion of recruitment [4, 8]. The analytic solutions provided in “Correlation models” section are based on some simple, but we believe realistic and useful, models of recruitment into the study and the serial correlation between the early and final outcome measures reported during participant follow-up. Although in general the correlations may be unknown at the planning stage, we can speculate on the likely correlation structure. In an analogous way to what we might do for variances in a conventional trial. At some arbitrarily selected point during recruitment we will not (in general) know the exact number of participants recruited or the number of participants ($N0_{r}$ and $N1_{r}$) with data at each follow-up occasion *r*. However, we can speculate on the likely recruitment rates and therefore the likely number of participants providing follow-up data at any point during the trial. Given the above we can predict how $\text {var}(\beta _{s})$, and therefore information, will vary during the study and use this to motivate the choice and timings of the interim analyses. The models provide analytic expressions for information accrual that can be routinely used at the planning stage of a putative trial, based on some modest assumptions about the likely number of outcomes and when they might occur and the expected recruitment patterns. Numerical simulations show that these models behave sensibly (i.e. in a manner that we would expect) and allow us to explore a range of design options in a way that would have been considerably more difficult and time-consuming if we had to use the previously described method of simulating individual trial participant data.

### Supplementary information

**Additional file 1: Figure S1.** The feasible region of $\mathrm V_{\mathrm s}^{\mathrm{unif}}$ for s = 2 (shaded areas), bounded above by the maximum and below by the minimum, for correlations in the range 0 ≤ α < 1 and equal group sizes (ϕ = 0.5) for the decreasing, fixed and increasing rate recruitment models with lines for the setting where the time-points are given by dr = 1+(r−1)/(s−1) (r = 1, 2) for [a] early (τ 01 = 0.15), [b] mid (τ 02 = 0.30) and [c] late (τ 03 = 0.45) interim analyses. **Figure S2.** The feasible region of $\mathrm V_{\mathrm s}^{\mathrm{unif}}$  for s = 3 (shaded areas), bounded above by the maximum and below by the minimum, for correlations in the range 0 ≤ α < 1 and equal group sizes (ϕ = 0.5) for the decreasing, fixed and increasing rate recruitment models with lines for the setting where the time-points are given by dr = 1+(r−1)/(s−1) (r = 1, 2, 3; i.e. equal spacing) for [a] early (τ 01 = 0.15), [b] mid (τ 02 = 0.30) and [c] late (τ 03 = 0.45) interim analyses. **Figure S3.** The empirical distribution (nsim = 10000) of Δ$\mathrm V_{\mathrm s}^{\mathrm{unif}}$  , the difference from the median value of $\mathrm V_{\mathrm s}^{\mathrm{unif}}$, with varying 1 < dr < 2 (r = 2) for s = 3, with shading showing quantiles 0-5%, 5-25%, 25-50%, 50-75%, 75-95% and 95-100%, for correlations in the range 0 ≤ α < 1 and equal group sizes (ϕ = 0.5) for [a] early (τ 01 = 0.15), [b] mid (τ 02 = 0.30) and [c] late (τ 03 = 0.45) interim analyses, for the (i) increasing, (ii) fixed and (iii) decreasing rate recruitment models. **Figure S4.** The feasible region of $\mathrm V_{\mathrm s}^{\mathrm{unif}}$ for s = 4 (shaded areas), bounded above by the maximum and below by the minimum, for correlations in the range 0 ≤ α < 1 and equal group sizes (ϕ = 0.5) for the decreasing, fixed and increasing rate recruitment models with lines for the setting where the time-points are given by dr = 1+(r−1)/(s−1) (r = 1, 2, 3, 4; i.e. equal spacing) for [a] early (τ 01 = 0.15), [b] mid (τ 02 = 0.30) and [c] late (τ 03 = 0.45) interim analyses. **Figure S5.** The empirical distribution (nsim = 10000) of Δ  , the difference from the median value of $\mathrm V_{\mathrm s}^{\mathrm{unif}}$ , with varying 1 < dr < 2 (r = 2, 3) for s = 4, with shading showing quantiles 0-5%, 5-25%, 25-50%, 50-75%, 75-95% and 95-100%, for correlations in the range 0 ≤ α < 1 and equal group sizes (ϕ = 0.5) for [a] early (τ 01 = 0.15), [b] mid (τ 02 = 0.30) and [c] late (τ 03 = 0.45) interim analyses, for the (i) increasing, (ii) fixed and (iii) decreasing rate recruitment models. **Figure S6.** The feasible region of $\mathrm V_{\mathrm s}^{\mathrm{unif}}$ for s = 5 (shaded areas), bounded above by the maximum and below by the minimum, for correlations in the range 0 ≤ α < 1 and equal group sizes (ϕ = 0.5) for the decreasing, fixed and increasing rate recruitment models with lines for the setting where the time-points are given by dr = 1+(r−1)/(s−1) (r = 1, 2, 3, 4, 5; i.e. equal spacing) for [a] early (τ 01 = 0.15), [b] mid (τ 02 = 0.30) and [c] late (τ 03 = 0.45) interim analyses.**Figure S7.** The empirical distribution (nsim = 10000) of Δ $\mathrm V_{\mathrm s}^{\mathrm{unif}}$, the difference from the median value of $\mathrm V_{\mathrm s}^{\mathrm{unif}}$ , with varying 1 < dr < 2 (r = 2, 3, 4) for s = 5, with shading showing quantiles 0-5%, 5-25%, 25-50%, 50-75%, 75-95% and 95-100%, for correlations in the range 0 ≤ α < 1 and equal group sizes (ϕ = 0.5) for [a] early (τ 01 = 0.15), [b] mid (τ 02 = 0.30) and [c] late (τ 03 = 0.45) interim analyses, for the (i) increasing, (ii) fixed and (iii) decreasing rate recruitment models. **Figure S8.** The feasible region of $\mathrm V_{\mathrm s}^{\mathrm{unif}}$ for s = 6 (shaded areas), bounded above by the maximum and below by the minimum, for correlations in the range 0 ≤ α < 1 and equal group sizes (ϕ = 0.5) for the decreasing, fixed and increasing rate recruitment models with lines for the setting where the time-points are given by dr = 1+(r−1)/(s−1) (r = 1, 2, 3, 4, 5, 6; i.e. equal spacing) for [a] early (τ 01 = 0.15), [b] mid (τ 02 = 0.30) and [c] late (τ 03 = 0.45) interim analyses. **Figure S9.** The empirical distribution (nsim = 10000) of Δ$\mathrm V_{\mathrm s}^{\mathrm{unif}}$  , the difference from the median value of $\mathrm V_{\mathrm s}^{\mathrm{unif}}$ , with varying 1 < dr < 2 (r = 2, 3, 4, 5) for s = 6, with shading showing quantiles 0-5%, 5-25%, 25-50%, 50-75%, 75-95% and 95-100%, for correlations in the range 0 ≤ α < 1 and equal group sizes (ϕ = 0.5) for [a] early (τ 01 = 0.15), [b] mid (τ 02 = 0.30) and [c] late (τ 03 = 0.45) interim analyses, for the (i) increasing, (ii) fixed and (iii) decreasing rate recruitment models.**Figure S10.** The feasible region of Vexp s for s = 2 (shaded areas), bounded above by the maximum and below by the minimum, for correlations in the range 0 ≤ γ < 1 and equal group sizes (ϕ = 0.5) for the decreasing, fixed and increasing rate recruitment models with lines for the setting where the time-points are given by dr = 1+(r−1)/(s−1) (r = 1, 2) for [a] early (τ 01= 0.15), [b] mid (τ 02 = 0.30) and [c] late (τ 03 = 0.45) interim analyses. **Figure S11. **The feasible region of Vexp s for s = 3 (shaded areas), bounded above by the maximum and below by the minimum, for correlations in the range 0 ≤ γ < 1 and equal group sizes (ϕ = 0.5) for the decreasing, fixed and increasing rate recruitment models with lines for the setting where the time-points are given by dr = 1+(r−1)/(s−1) (r = 1, 2, 3; i.e. equal spacing) for [a] early (τ 01 = 0.15), [b] mid (τ 02 = 0.30) and [c] late (τ 03 = 0.45) interim analyses. **Figure S12.** The empirical distribution (nsim = 10000) of ΔVexp s , the difference from the median value of Vexp s , with varying 1 < dr < 2 (r = 2) for s = 3, with shading showing quantiles 0-5%, 5-25%, 25-50%, 50-75%, 75-95% and 95-100%, for correlations in the range 0 ≤ γ < 1 and equal group sizes (ϕ = 0.5) for [a] early (τ 01 = 0.15), [b] mid (τ 02 = 0.30) and [c] late (τ 03 = 0.45) interim analyses, for the (i) increasing, (ii) fixed and (iii) decreasing rate recruitment models. **Figure S13.** The feasible region of Vexp s for s = 4 (shaded areas), bounded above by the maximum and below by the minimum, for correlations in the range 0 ≤ γ < 1 and equal group sizes (ϕ = 0.5) for the decreasing, fixed and increasing rate recruitment models with lines for the setting where the time-points are given by dr = 1+(r−1)/(s−1) (r = 1, 2, 3, 4; i.e. equal spacing) for [a] early (τ 01 = 0.15), [b] mid (τ 02 = 0.30) and [c] late (τ 03 = 0.45) interim analyses. **Figure S14.** The empirical distribution (nsim = 10000) of ΔVexp s , the difference from the median value of Vexp s , with varying 1 < dr < 2 (r = 2, 3) for s = 4, with shading showing quantiles 0-5%, 5-25%, 25-50%, 50-75%, 75-95% and 95-100%, for correlations in the range 0 ≤ γ < 1 and equal group sizes (ϕ = 0.5) for [a] early (τ 01 = 0.15), [b] mid (τ 02 = 0.30) and [c] late (τ 03 = 0.45) interim analyses, for the (i) increasing, (ii) fixed and (iii) decreasing rate recruitment models. **Figure S15**. The feasible region of Vexp s for s = 5 (shaded areas), bounded above by the maximum and below by the minimum, for correlations in the range 0 ≤ γ < 1 and equal group sizes (ϕ = 0.5) for the decreasing, fixed and increasing rate recruitment models with lines for the setting where the time-points are given by dr = 1+(r−1)/(s−1) (r = 1, 2, 3, 4, 5; i.e. equal spacing) for [a] early (τ 01 = 0.15), [b] mid (τ 02 = 0.30) and [c] late (τ 03 = 0.45) interim analyses. **Figure S16**. The empirical distribution (nsim = 10000) of ΔVexp s , the difference from the median value of Vexp s , with varying 1 < dr < 2 (r = 2, 3, 4) for s = 5, with shading showing quantiles 0-5%, 5-25%, 25-50%, 50-75%, 75-95% and 95-100%, for correlations in the range 0 ≤ γ < 1 and equal group sizes (ϕ = 0.5) for [a] early (τ 01 = 0.15), [b] mid (τ 02 = 0.30) and [c] late (τ 03 = 0.45) interim analyses, for the (i) increasing, (ii) fixed and (iii) decreasing rate recruitment models. **Figure S17.** The feasible region of Vexps for s = 6 (shaded areas), bounded above by the maximum and below by the minimum, for correlations in the range 0 ≤ γ < 1 and equal group sizes (ϕ = 0.5) for the decreasing, fixed and increasing rate recruitment models with lines for the setting where the time-points are given by dr = 1+(r−1)/(s−1) (r = 1, 2, 3, 4, 5, 6; i.e. equal spacing) for [a] early (τ 01 = 0.15), [b] mid (τ 02 = 0.30) and [c] late (τ 03 = 0.45) interim analyses. **Figure S18.** The empirical distribution (nsim = 10000) of ΔVexp s , the difference from the median value of Vexp s , with varying 1 < dr < 2 (r = 2, 3, 4, 5) for s = 6, with shading showing quantiles 0-5%, 5-25%, 25-50%, 50-75%, 75-95% and 95-100%, for correlations in the range 0 ≤ γ < 1 and equal group sizes (ϕ = 0.5) for [a] early (τ 01 = 0.15), [b] mid (τ 02 = 0.30) and [c] late (τ 03 = 0.45) interim analyses, for the (i) increasing, (ii) fixed and (iii) decreasing rate recruitment models.

**Additional file 1: Appendix A.** A.1 Uniform correlation model. A.2 Exponential correlation model. A.3 Partial derivatives of Vexps. A.4 Recruitment and follow-up models. Table A1 Times (t1, t2 and t3) for early τ0(t1) = 0.15, mid τ0(t2) = 0.30 and late τ0(t3) = 0.45 interim analyses, for increasing, fixed and decreasing rate recruitment models.

## Data Availability

Not applicable. as no data or materials were used in this research.

## Code availability

The code used to generate the numerical examples is available on request from the corresponding author (NP). Work is ongoing to develop a package of R functions to allow simple implementation of the models described.

## References

[1] Hatfield I, Allison A, Flight L, Julious SA, Dimairo M (2016). Adaptive designs undertaken in clinical research: a review of registered clinical trials. Trials..

[2] Jennison C, Turnbull BW (2000). Group sequential methods with applications to clinical trials.

[3] Dimairo M, Boote J, Julious SA, Nicholl JP, Todd S (2015). Missing steps in a staircase: a qualitative study of the perspectives of key stakeholders on the use of adaptive designs in confirmatory trials. Trials..

[4] Parsons NR, Stallard N, Parsons H, Haque A, Underwood M, Mason J, et al. Group sequential designs in pragmatic trials: feasibility and assessment of utility using data from a number of recent surgical RCTs. BMC Med Res Methodol. 2022;22(1). 10.1186/s12874-022-01734-2. https://www.scopus.com/inward/record.uri?eid=2-s2.0-85139109334 &doi=10.1186%2fs12874-022-01734-2 &partnerID=40 &md5=39107fa728feb689a7f31f5569cd006e. 10.1186/s12874-022-01734-2 PMC9526271 36183085

[5] Roland M, Torgerson DJ (1998). What are pragmatic trials?. BMJ..

[6] Ford I, Norrie J (2016). Pragmatic trials. N Engl J Med..

[7] Metcalfe A, Parsons H, Parsons N, Brown J, Fox J, Gemperle Mannion E (2022). Subacromial balloon spacer for irreparable rotator cuff tears of the shoulder (START:REACTS): a group-sequential, double-blind, multicentre randomised controlled trial. Lancet..

[8] Parsons N, Stallard N, Parsons H, Wells P, Underwood M, Mason J, et al. An adaptive two-arm clinical trial using early endpoints to inform decision making: design for a study of sub-acromial spacers for repair of rotator cuff tendon tears. Trials. 2019;20(1):694. 10.1186/s13063-019-3708-6. https://www.ncbi.nlm.nih.gov/pubmed/31815651 10.1186/s13063-019-3708-6 PMC6902495 31815651

[9] Barnard KD, Dent L, Cook A. A systematic review of models to predict recruitment to multicentre clinical trials. BMC Med Res Methodol. 2010;10:63. 10.1186/1471-2288-10-63. 10.1186/1471-2288-10-63 PMC2908107 20604946

[10] Galbraith S, Marschner IC (2003). Interim analysis of continuous long-term endpoints in clinical trials with longitudinal outcomes. Stat Med..

[11] Jennison C, Turnbull BW (1997). Group-sequential analysis incorporating covariate information. J Am Stat Assoc..

[12] Van Lancker K, Vandebosch A, Vansteelandt S (2020). Improving interim decisions in randomized trials by exploiting information on short-term endpoints and prognostic baseline covariates. Pharm Stat..

[13] Viele K, McGlothlin A, Broglio K (2016). Interpretation of clinical trials that stopped early. JAMA..

[14] Liu A, Hall W (1999). Unbiased estimation following a group sequential test. Biometrika..

[15] Todd S, Whitehead J, Facey KM. Point and interval estimation following a sequential clinical trial. Biometrika. 1996 06;83(2):453–461.

[16] Stallard N (2010). A confirmatory seamless Phase II/III clinical trial design incorporating short-term endpoint information. Stat Med..

[17] Engel B, Walstra P. Increasing precision or reducing expense in regression experiments by using information from a concomitant variable. Biometrics. 1991;47(1):13–20. 10.2307/2532491. https://www.jstor.org/stable/2532491

[18] Spiessens B, Lesaffre E, Verbeke G, Kim K, DeMets DL (2000). An overview of group sequential methods in longitudinal clinical trials. Stat Methods Med Res..

[19] Spiessens B, Lesaffre E, Verbeke G (2003). A comparison of group sequential methods for binary longitudinal data. Stat Med..

[20] Kim K, Anastasios AT (2020). Independent increments in group sequential tests: a review. Stat Oper Res Trans..

[21] Qian T, Rosenblum M, Qiu H. Improving power in group sequential, randomized trials by adjusting for prognostic baseline variables and shortterm outcomes. Johns Hopkins University, Dept of Biostatistics Working Papers, Working paper 285. 2016;5(5):5.

[22] Diggle P, Diggle P (2002). Analysis of longitudinal data.

[23] R Core Team. R: A Language and Environment for Statistical Computing. Vienna; 2022. https://www.R-project.org/. Accessed 3 Nov 2023.

[24] Pinheiro JC, Bates D (2009). Mixed-Effects Models in S and S-PLUS.

[25] Lan KKG, Reboussin DM, DeMets DL (1994). Information and information fractions for design and sequential monitoring of clinical trials. Commun Stat Theory Methods..

[26] Anderson K. gsDesign: Group Sequential Design. 2021. https://CRAN.R-project.org/package=gsDesign. Accessed 3 Nov 2023.

[27] Metcalfe A, Gemperle Mannion E, Parsons H, Brown J, Parsons N, Fox J, et al. Protocol for a randomised controlled trial of Subacromial spacer for Tears Affecting Rotator cuff Tendons: a Randomised, Efficient, Adaptive Clinical Trial in Surgery (START:REACTS). BMJ Open. 2020;10(5): e036829. 10.1136/bmjopen-2020-036829. https://www.ncbi.nlm.nih.gov/pubmed/32444433 10.1136/bmjopen-2020-036829 PMC7247380 32444433

