# Supplementary material for: Group sequential designs for pragmatic clinical trials with early outcomes: methods and guidance for planning and implementation
Source: BMC Med Res Methodol. 2024 Feb 16;24:42. doi: 10.1186/s12874-024-02174-w (PMC10870612; doi:10.1186/s12874-024-02174-w)
Supplement: Supplementary file 1 — Additional file 1: Figure S1. The feasible region of $\mathrm V_{\mathrm s}^{\mathrm{unif}}$Vsunif for s = 2 (shaded areas), bounded above by the maximum and below by the minimum, for correlations in the range 0 ≤ α < 1 and equal group sizes (ϕ = 0.5) for the decreasing, fixed and increasing rate recruitment models with lines for the setting where the time-points are given by dr = 1+(r−1)/(s−1) (r = 1, 2) for [a] early (τ 01 = 0.15), [b] mid (τ 02 = 0.30) and [c] late (τ 03 = 0.45) interim analyses. Figure S2. The feasible region of $\mathrm V_{\mathrm s}^{\mathrm{unif}}$Vsunif for s = 3 (shaded areas), bounded above by the maximum and below by the minimum, for correlations in the range 0 ≤ α < 1 and equal group sizes (ϕ = 0.5) for the decreasing, fixed and increasing rate recruitment models with lines for the setting where the time-points are given by dr = 1+(r−1)/(s−1) (r = 1, 2, 3; i.e. equal spacing) for [a] early (τ 01 = 0.15), [b] mid (τ 02 = 0.30) and [c] late (τ 03 = 0.45) interim analyses. Figure S3. The empirical distribution (nsim = 10000) of Δ$\mathrm V_{\mathrm s}^{\mathrm{unif}}$Vsunif , the difference from the median value of [file 12874_2024_2174_MOESM1_ESM.pdf]

Performance characteristics for group sequential designs with multiple early outcomes, varying correlation and recruitment rate models and interim analysis timings

Nick R Parsons[1], Joydeep Basu[1], and Nigel Stallard[1]

[1]Warwick Clinical Trials Unit (WCTU), Warwick Medical School, University of Warwick, CV4 7AL, Coventry, UK

November 3, 2023

1 Uniform correlation model

1.1 Two outcomes (s=2)

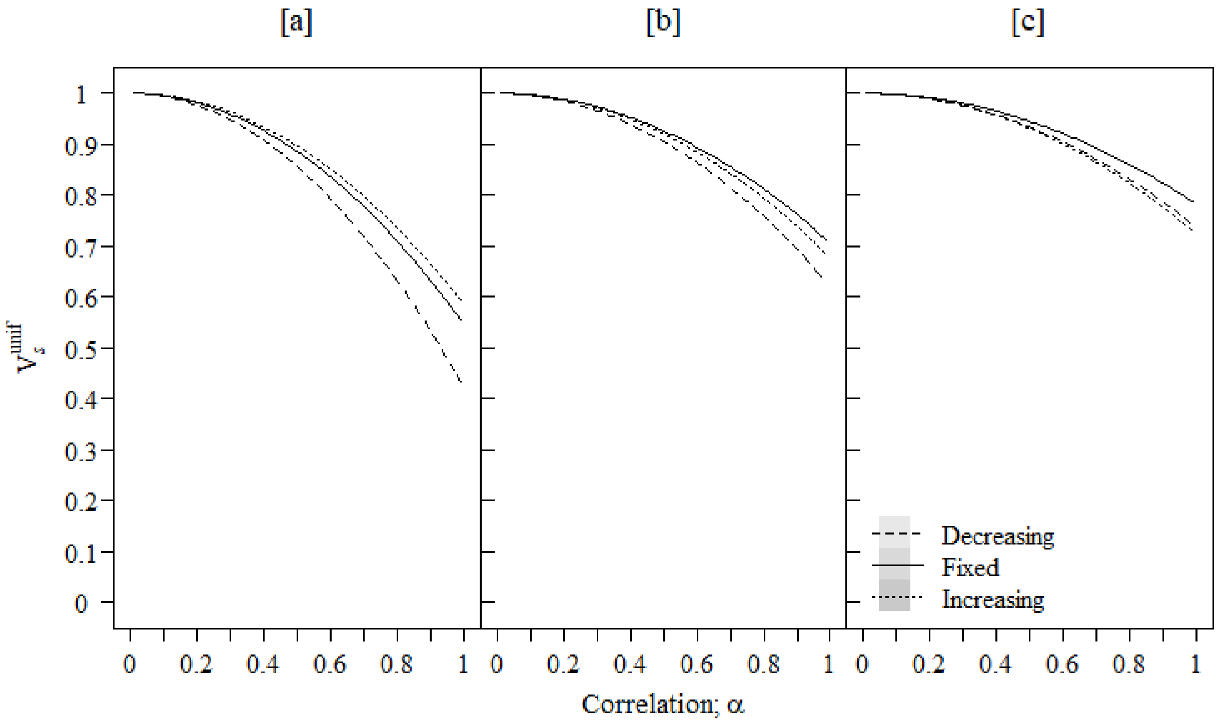

Figure S1: The feasible region of V_s^{unif} for $s = 2$ (shaded areas), bounded above by the maximum and below by the minimum, for correlations in the range $0 \leq \alpha < 1$ and equal group sizes ($\phi = 0.5$) for the decreasing, fixed and increasing rate recruitment models with lines for the setting where the time-points are given by $d_r = 1+(r-1)/(s-1)$ ($r = 1, 2$) for [a] early ($\tau_{0_1} = 0.15$), [b] mid ($\tau_{0_2} = 0.30$) and [c] late ($\tau_{0_3} = 0.45$) interim analyses.

1.2 Three outcomes (s=3)

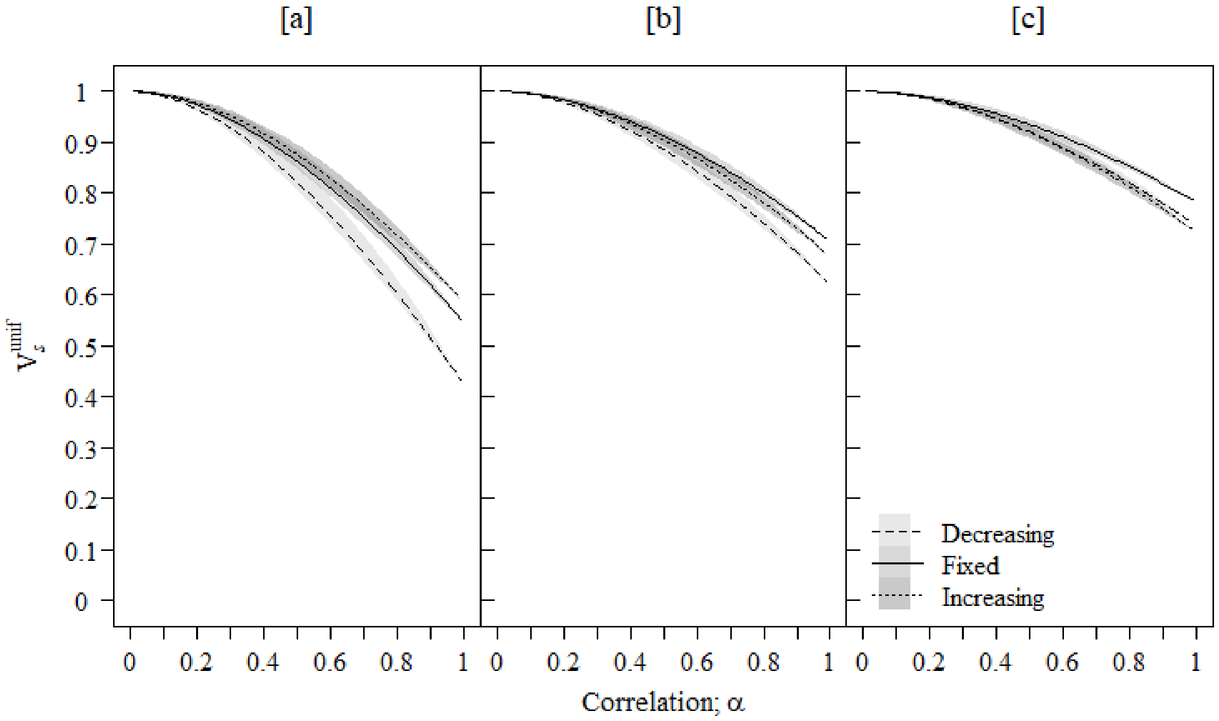

Figure S2: The feasible region of V_s^{unif} for $s = 3$ (shaded areas), bounded above by the maximum and below by the minimum, for correlations in the range $0 \leq \alpha < 1$ and equal group sizes ($\phi = 0.5$) for the decreasing, fixed and increasing rate recruitment models with lines for the setting where the time-points are given by $d_r = 1+(r-1)/(s-1)$ ($r = 1, 2, 3$; i.e. equal spacing) for [a] early ($\tau 0_1 = 0.15$), [b] mid ($\tau 0_2 = 0.30$) and [c] late ($\tau 0_3 = 0.45$) interim analyses.

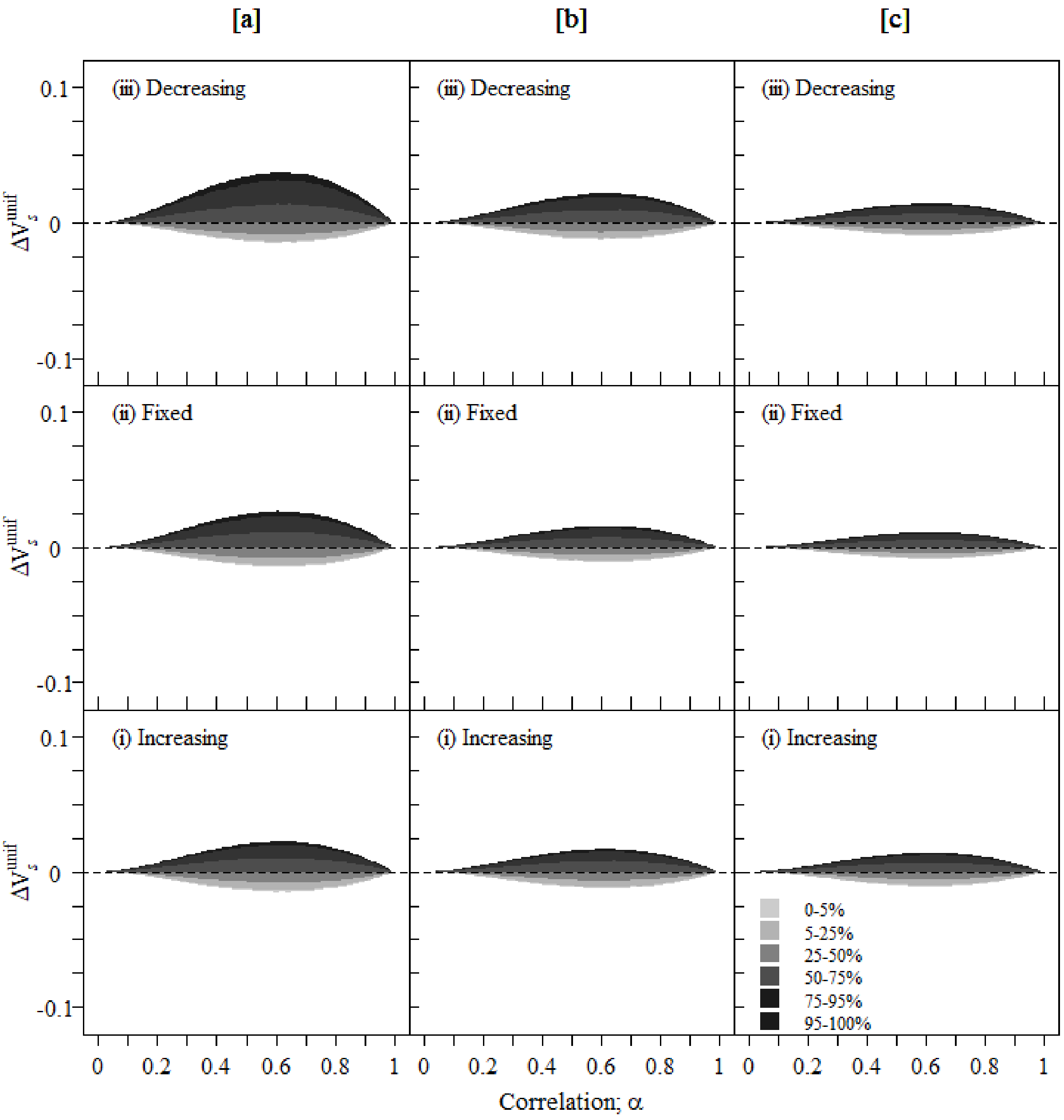

Figure S3: The empirical distribution ($n_{sim} = 10000$) of $\Delta \mathrm{V}_s^{\mathrm{unif}}$, the difference from the median value of $\mathrm{V}_s^{\mathrm{unif}}$, with varying $1 < d_r < 2$ ($r = 2$) for $s = 3$, with shading showing quantiles 0-5%, 5-25%, 25-50%, 50-75%, 75-95% and 95-100%, for correlations in the range $0 \leq \alpha < 1$ and equal group sizes ($\phi = 0.5$) for [a] early ($\tau_{0_1} = 0.15$), [b] mid ($\tau_{0_2} = 0.30$) and [c] late ($\tau_{0_3} = 0.45$) interim analyses, for the (i) increasing, (ii) fixed and (iii) decreasing rate recruitment models.

1.3 Four outcomes (s=4)

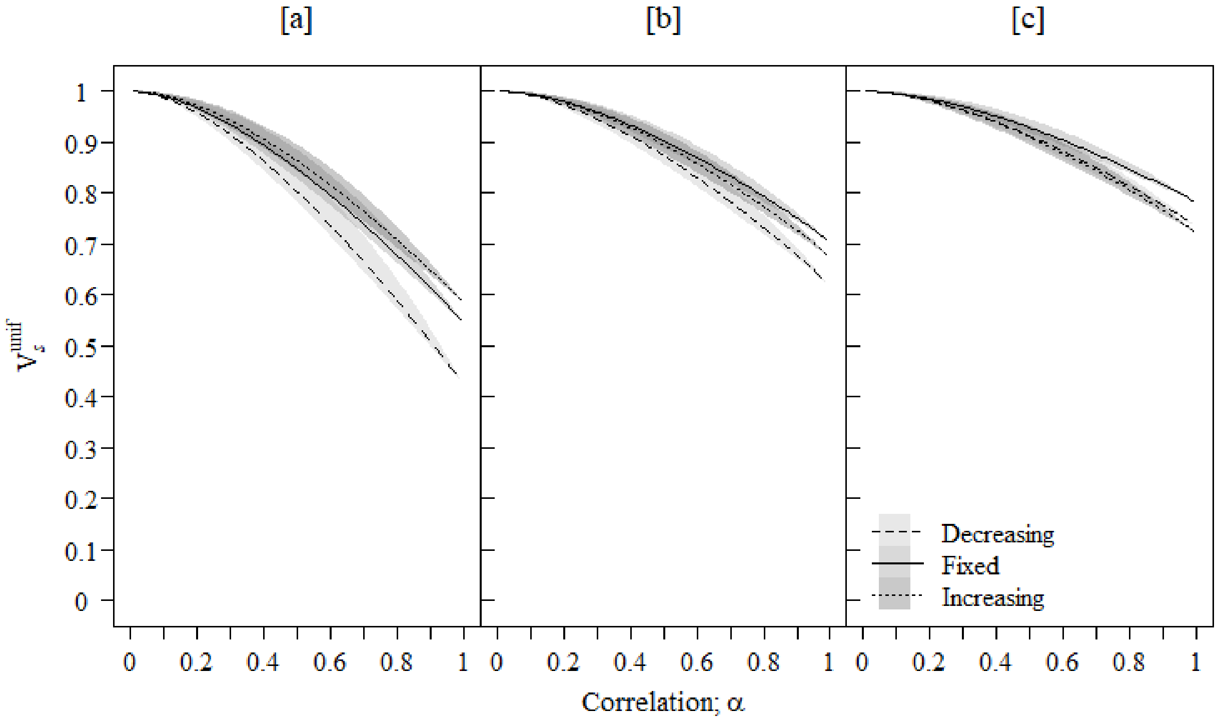

Figure S4: The feasible region of V_s^{unif} for $s = 4$ (shaded areas), bounded above by the maximum and below by the minimum, for correlations in the range $0 \leq \alpha < 1$ and equal group sizes ($\phi = 0.5$) for the decreasing, fixed and increasing rate recruitment models with lines for the setting where the time-points are given by $d_r = 1+(r-1)/(s-1)$ ($r = 1, 2, 3, 4$; i.e. equal spacing) for [a] early ($\tau_{0_1} = 0.15$), [b] mid ($\tau_{0_2} = 0.30$) and [c] late ($\tau_{0_3} = 0.45$) interim analyses.

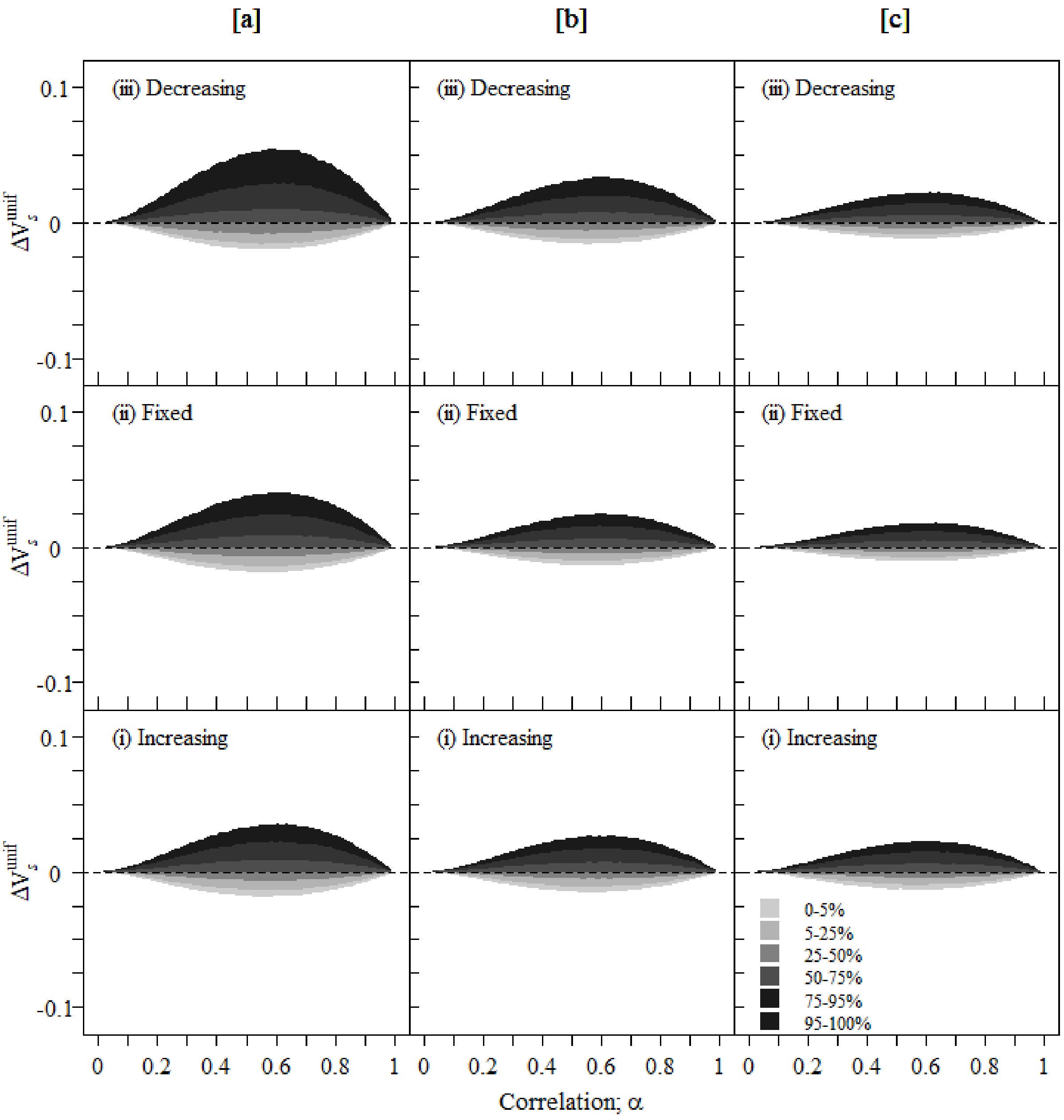

Figure S5: The empirical distribution ($n_{sim} = 10000$) of ΔV_s^{unif}, the difference from the median value of V_s^{unif}, with varying $1 < d_r < 2$ ($r = 2, 3$) for $s = 4$, with shading showing quantiles 0-5%, 5-25%, 25-50%, 50-75%, 75-95% and 95-100%, for correlations in the range $0 \leq \alpha < 1$ and equal group sizes ($\phi = 0.5$) for [a] early ($\tau_{0_1} = 0.15$), [b] mid ($\tau_{0_2} = 0.30$) and [c] late ($\tau_{0_3} = 0.45$) interim analyses, for the (i) increasing, (ii) fixed and (iii) decreasing rate recruitment models.

1.4 Five outcomes (s=5)

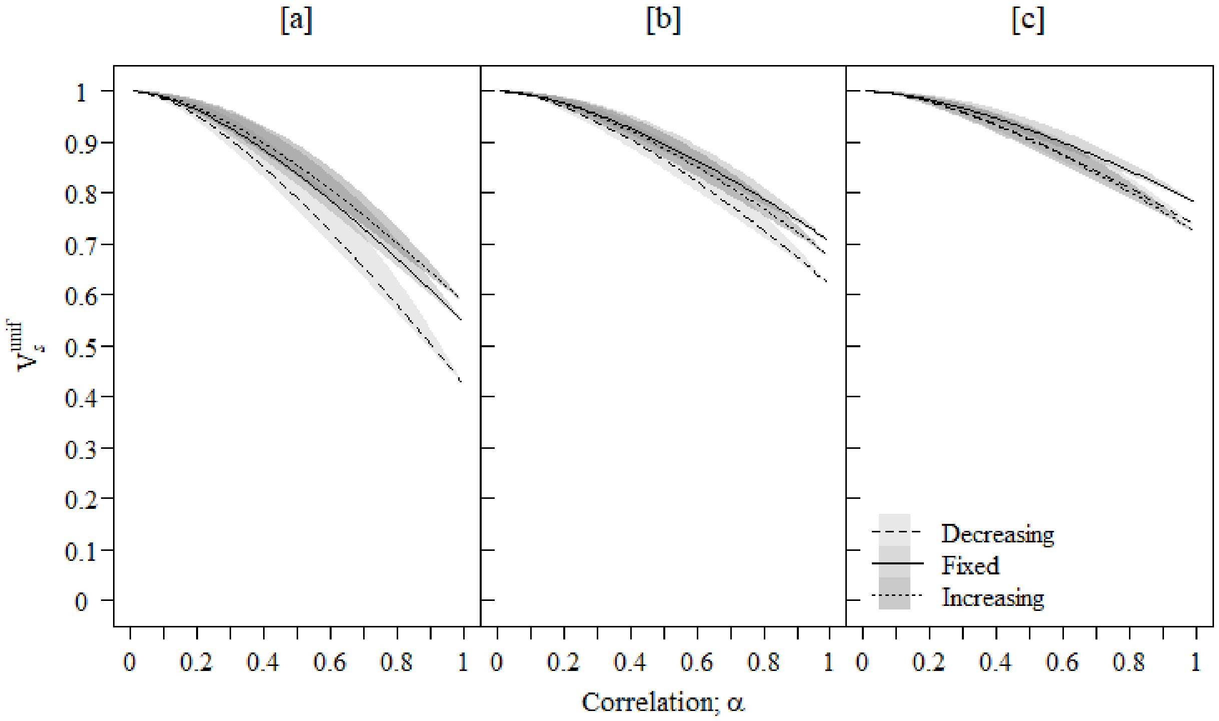

Figure S6: The feasible region of V_s^{unif} for $s = 5$ (shaded areas), bounded above by the maximum and below by the minimum, for correlations in the range $0 \leq \alpha < 1$ and equal group sizes ($\phi = 0.5$) for the decreasing, fixed and increasing rate recruitment models with lines for the setting where the time-points are given by $d_r = 1+(r-1)/(s-1)$ ($r = 1, 2, 3, 4, 5$; i.e. equal spacing) for [a] early ($\tau o_1 = 0.15$), [b] mid ($\tau o_2 = 0.30$) and [c] late ($\tau o_3 = 0.45$) interim analyses.

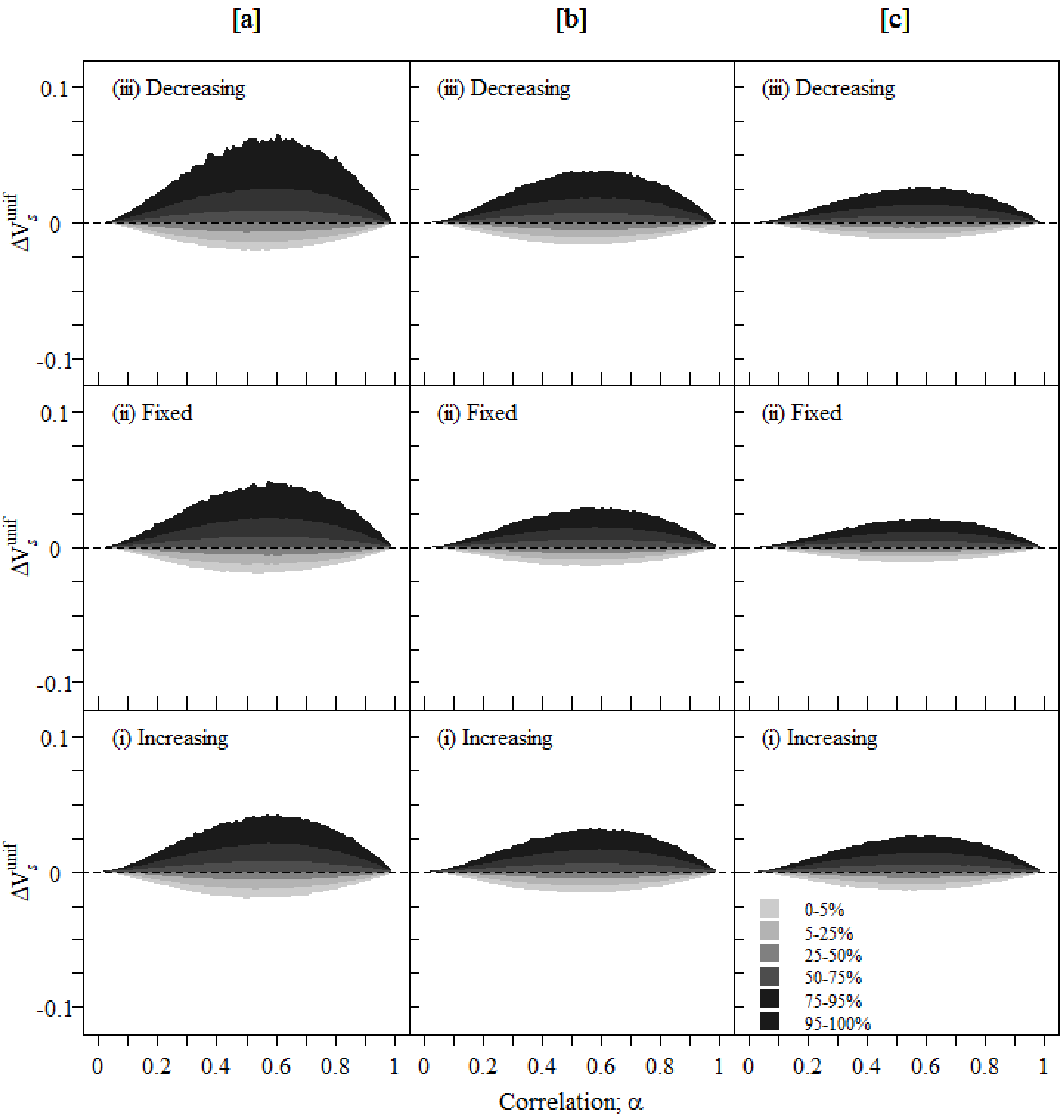

Figure S7: The empirical distribution ($n_{sim} = 10000$) of $\Delta \mathrm{V}_s^{\mathrm{unif}}$, the difference from the median value of $\mathrm{V}_s^{\mathrm{unif}}$, with varying $1 < d_r < 2$ ($r = 2, 3, 4$) for $s = 5$, with shading showing quantiles 0-5%, 5-25%, 25-50%, 50-75%, 75-95% and 95-100%, for correlations in the range $0 \leq \alpha < 1$ and equal group sizes ($\phi = 0.5$) for [a] early ($\tau_{0_1} = 0.15$), [b] mid ($\tau_{0_2} = 0.30$) and [c] late ($\tau_{0_3} = 0.45$) interim analyses, for the (i) increasing, (ii) fixed and (iii) decreasing rate recruitment models.

1.5 Six outcomes (s=6)

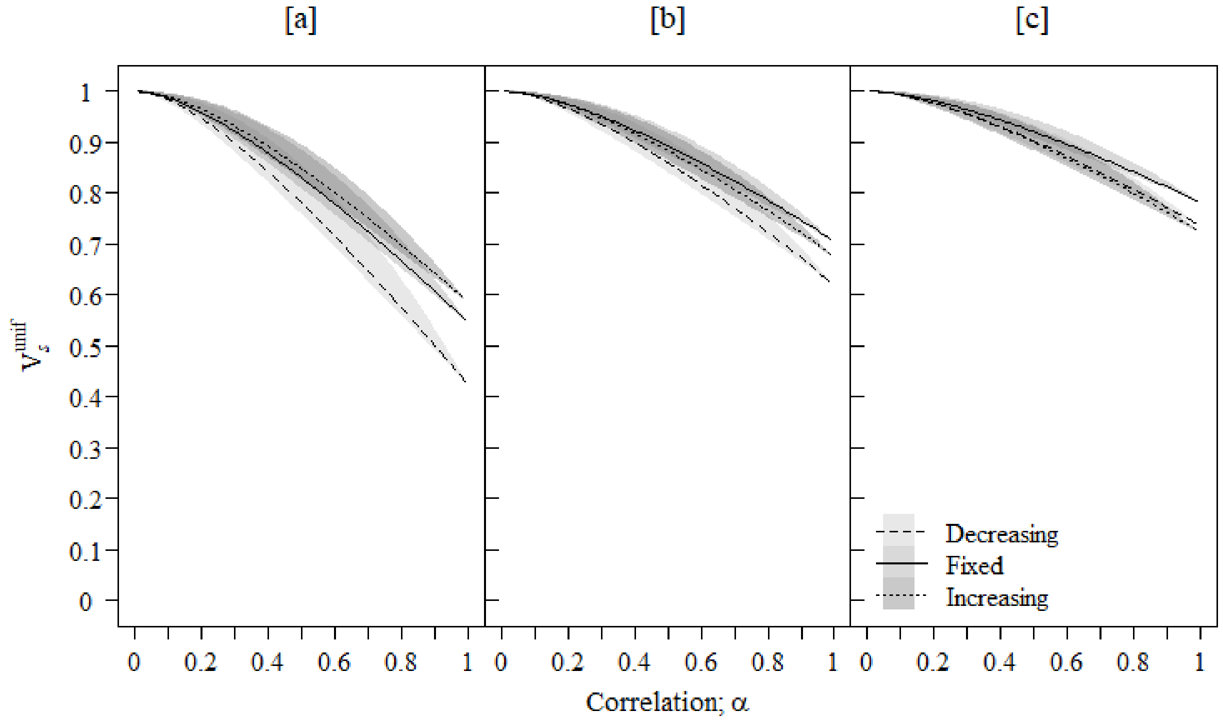

Figure S8: The feasible region of V_s^{unif} for $s = 6$ (shaded areas), bounded above by the maximum and below by the minimum, for correlations in the range $0 \leq \alpha < 1$ and equal group sizes ($\phi = 0.5$) for the decreasing, fixed and increasing rate recruitment models with lines for the setting where the time-points are given by $d_r = 1+(r-1)/(s-1)$ ($r = 1, 2, 3, 4, 5, 6$; i.e. equal spacing) for [a] early ($\tau_{0_1} = 0.15$), [b] mid ($\tau_{0_2} = 0.30$) and [c] late ($\tau_{0_3} = 0.45$) interim analyses.

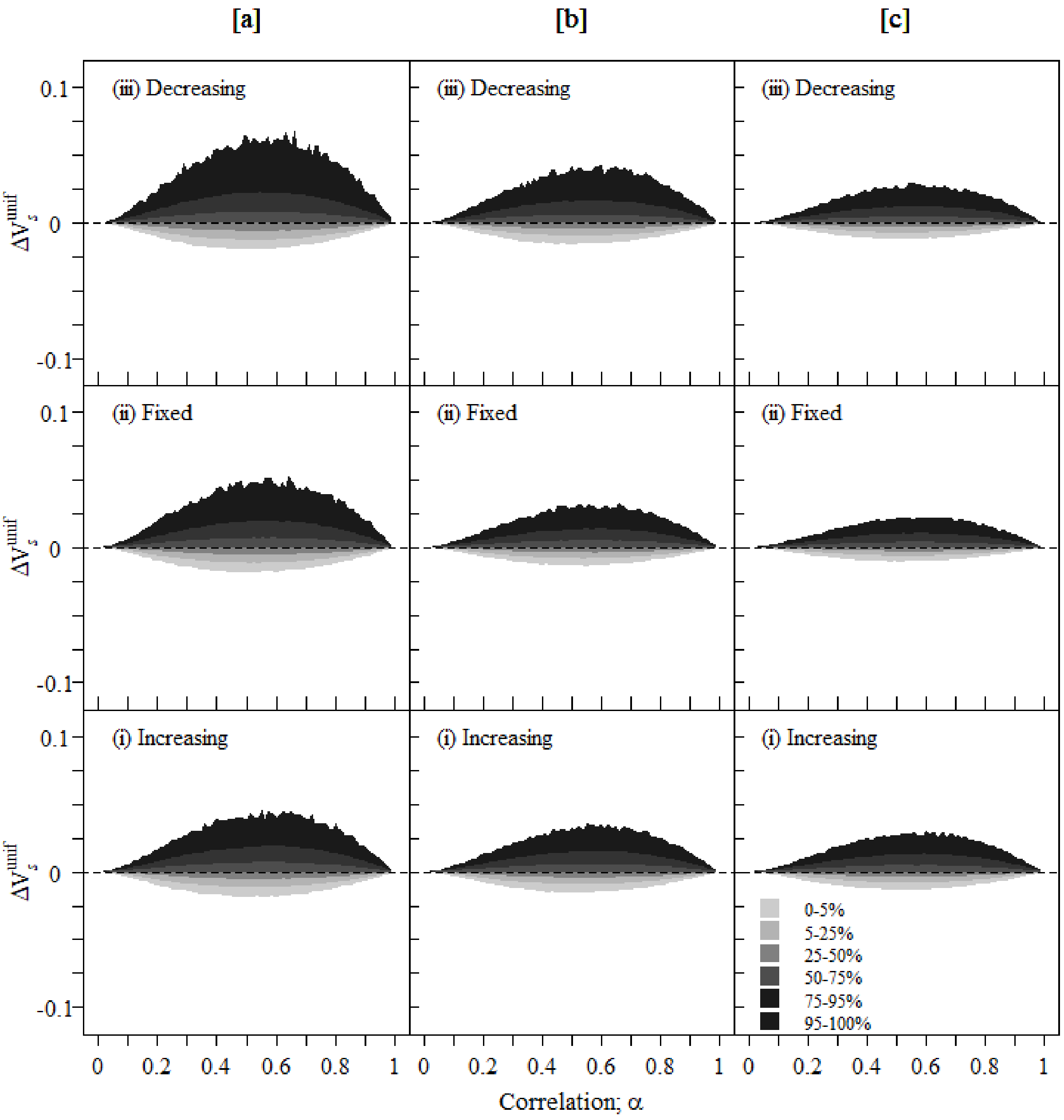

Figure S9: The empirical distribution ($n_{sim} = 10000$) of $\Delta \mathrm{V}_s^{\mathrm{unif}}$, the difference from the median value of $\mathrm{V}_s^{\mathrm{unif}}$, with varying $1 < d_r < 2$ ($r = 2, 3, 4, 5$) for $s = 6$, with shading showing quantiles 0-5%, 5-25%, 25-50%, 50-75%, 75-95% and 95-100%, for correlations in the range $0 \leq \alpha < 1$ and equal group sizes ($\phi = 0.5$) for [a] early ($\tau o_1 = 0.15$), [b] mid ($\tau o_2 = 0.30$) and [c] late ($\tau o_3 = 0.45$) interim analyses, for the (i) increasing, (ii) fixed and (iii) decreasing rate recruitment models.

2 Exponential correlation model

2.1 Two outcomes (s=2)

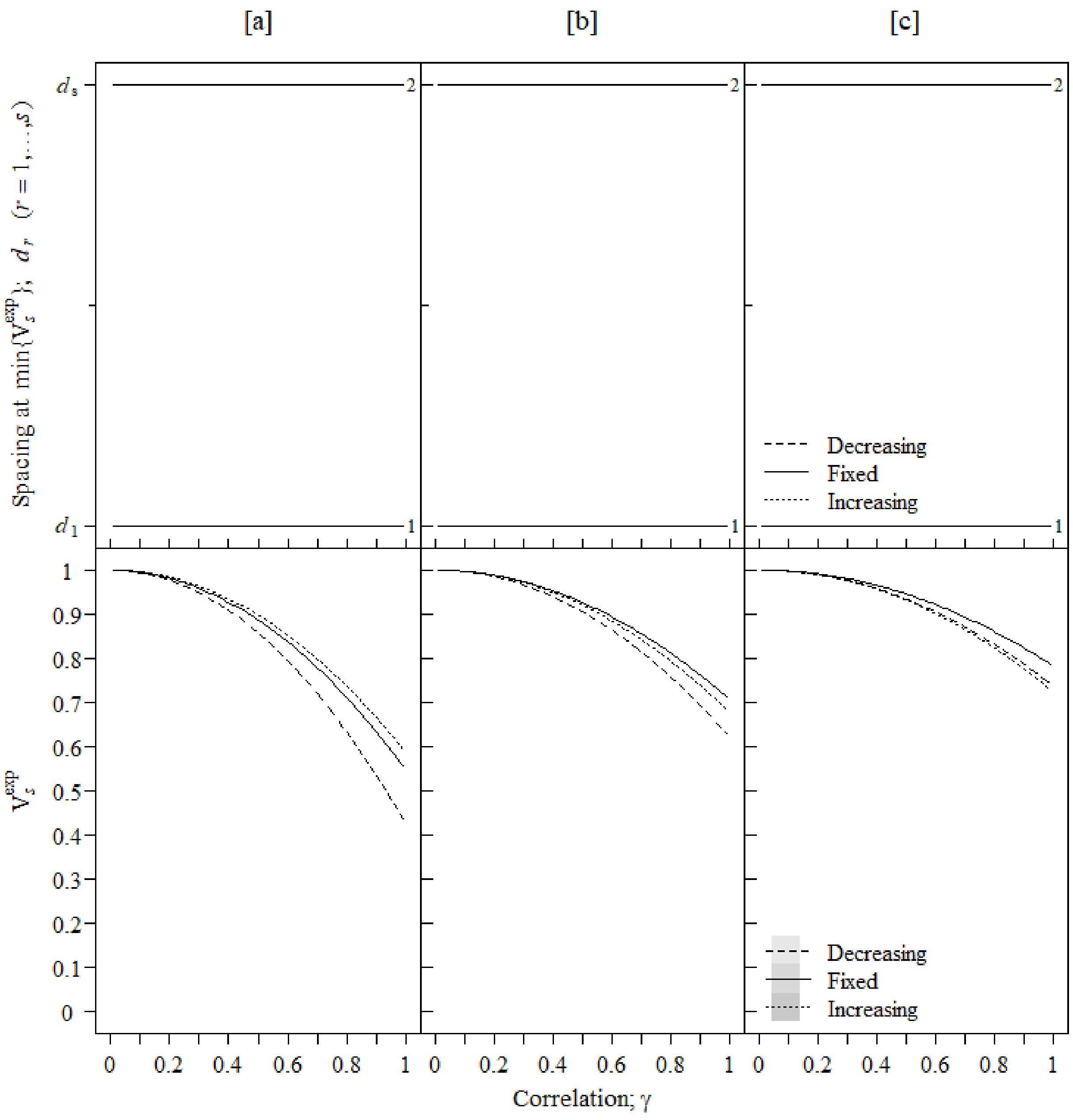

Figure S10: The feasible region of V_s^{exp} for $s = 2$ (shaded areas), bounded above by the maximum and below by the minimum, for correlations in the range $0 \leq \gamma < 1$ and equal group sizes ($\phi = 0.5$) for the decreasing, fixed and increasing rate recruitment models with lines for the setting where the time-points are given by $d_r = 1+(r-1)/(s-1)$ $(r = 1, 2)$ for [a] early ($\tau_{0_1} = 0.15$), [b] mid ($\tau_{0_2} = 0.30$) and [c] late ($\tau_{0_3} = 0.45$) interim analyses.

2.2 Three outcomes (s=3)

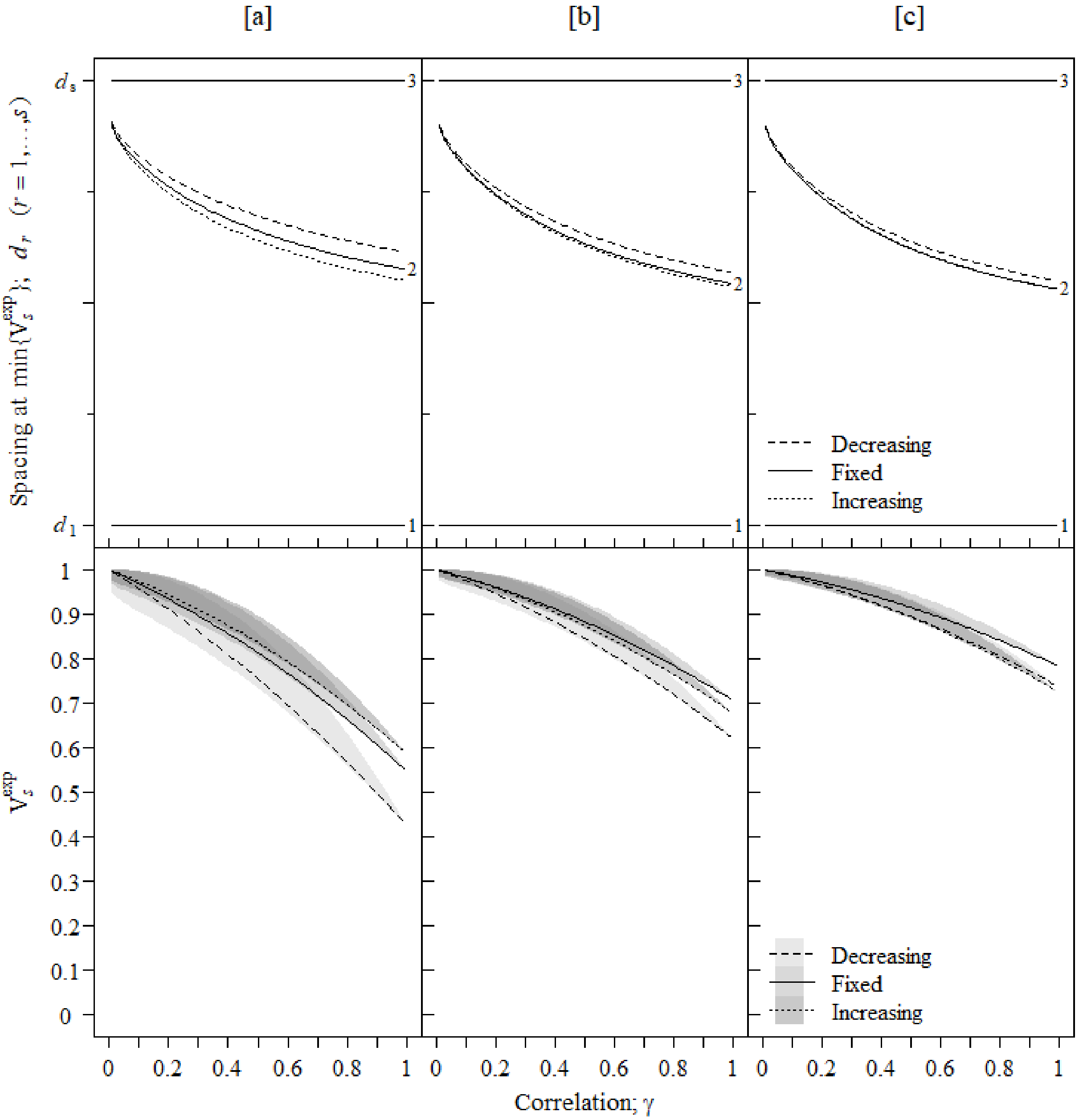

Figure S11: The feasible region of $\mathrm{V}_s^{\mathrm{exp}}$ for $s = 3$ (shaded areas), bounded above by the maximum and below by the minimum, for correlations in the range $0 \leq \gamma < 1$ and equal group sizes ($\phi = 0.5$) for the decreasing, fixed and increasing rate recruitment models with lines for the setting where the time-points are given by $d_r = 1+(r-1)/(s-1)$ ($r = 1, 2, 3$; i.e. equal spacing) for [a] early ($\tau_{0_1} = 0.15$), [b] mid ($\tau_{0_2} = 0.30$) and [c] late ($\tau_{0_3} = 0.45$) interim analyses.

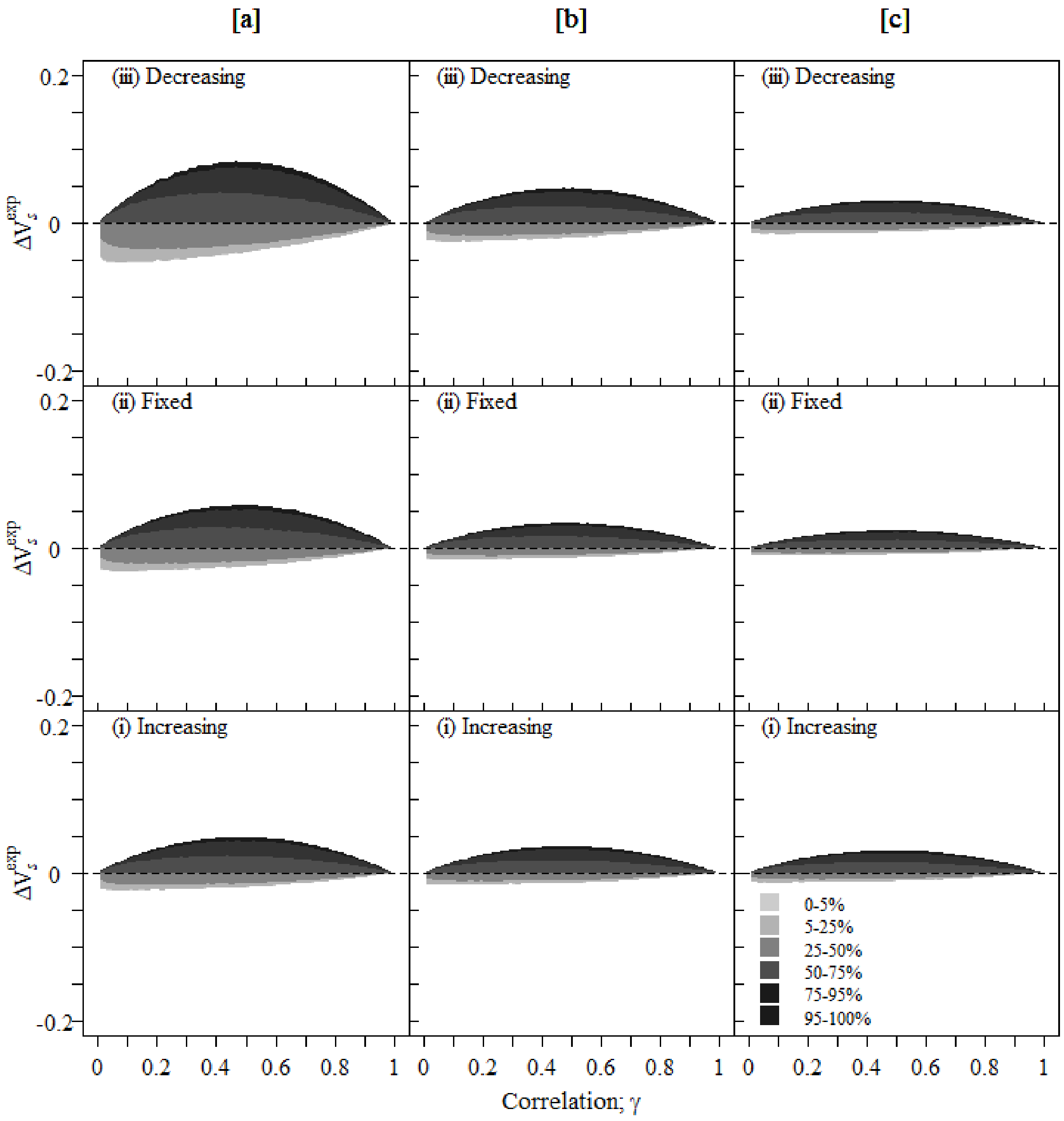

Figure S12: The empirical distribution ($n_{sim} = 10000$) of ΔV_s^{exp}, the difference from the median value of V_s^{exp}, with varying $1 < d_r < 2$ ($r = 2$) for $s = 3$, with shading showing quantiles 0-5%, 5-25%, 25-50%, 50-75%, 75-95% and 95-100%, for correlations in the range $0 \leq \gamma < 1$ and equal group sizes ($\phi = 0.5$) for [a] early ($\tau_{0_1} = 0.15$), [b] mid ($\tau_{0_2} = 0.30$) and [c] late ($\tau_{0_3} = 0.45$) interim analyses, for the (i) increasing, (ii) fixed and (iii) decreasing rate recruitment models.

2.3 Four outcomes (s=4)

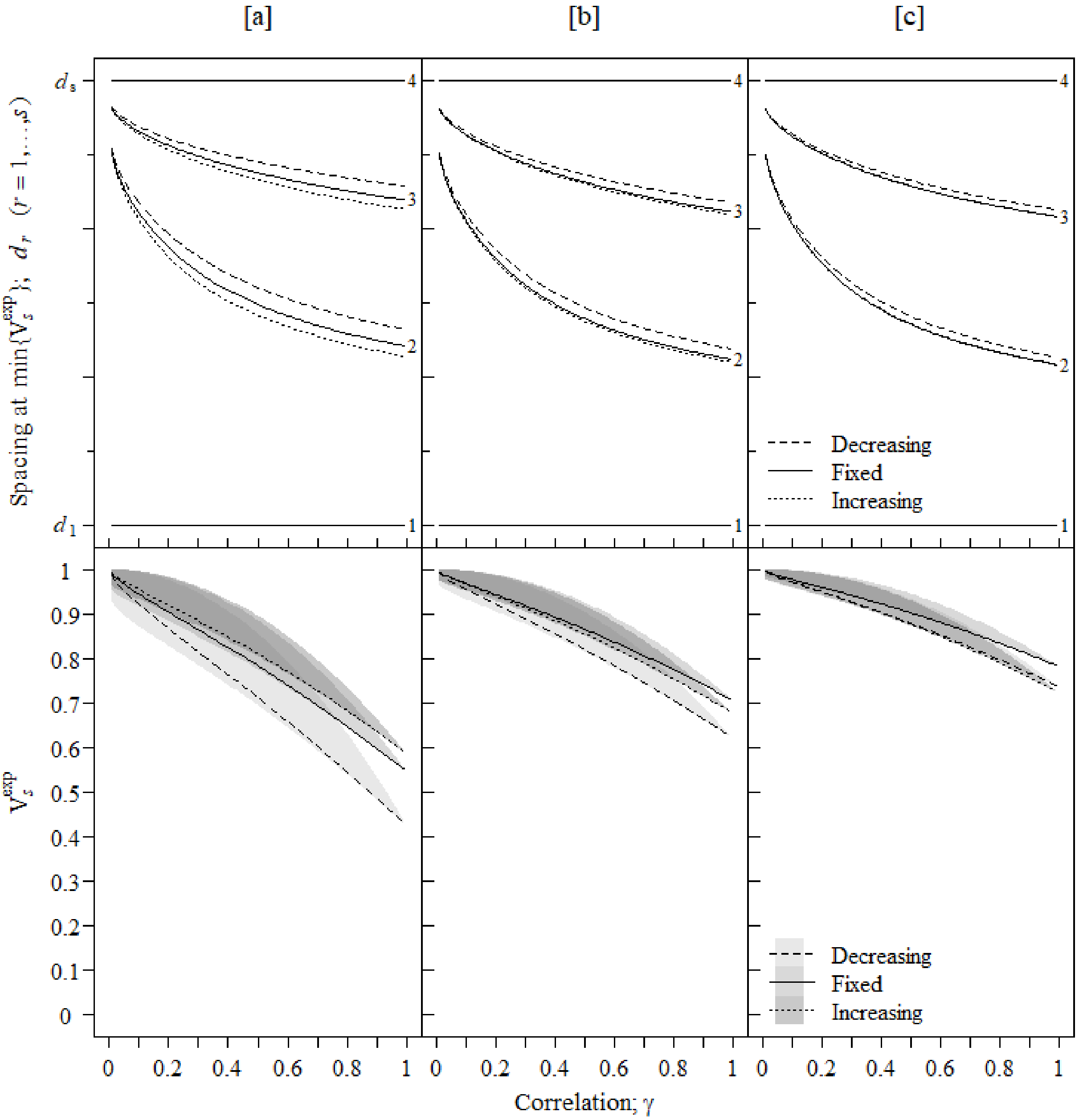

Figure S13: The feasible region of V_s^{exp} for $s = 4$ (shaded areas), bounded above by the maximum and below by the minimum, for correlations in the range $0 \leq \gamma < 1$ and equal group sizes ($\phi = 0.5$) for the decreasing, fixed and increasing rate recruitment models with lines for the setting where the time-points are given by $d_r = 1+(r-1)/(s-1)$ ($r = 1, 2, 3, 4$; i.e. equal spacing) for [a] early ($\tau_{0_1} = 0.15$), [b] mid ($\tau_{0_2} = 0.30$) and [c] late ($\tau_{0_3} = 0.45$) interim analyses.

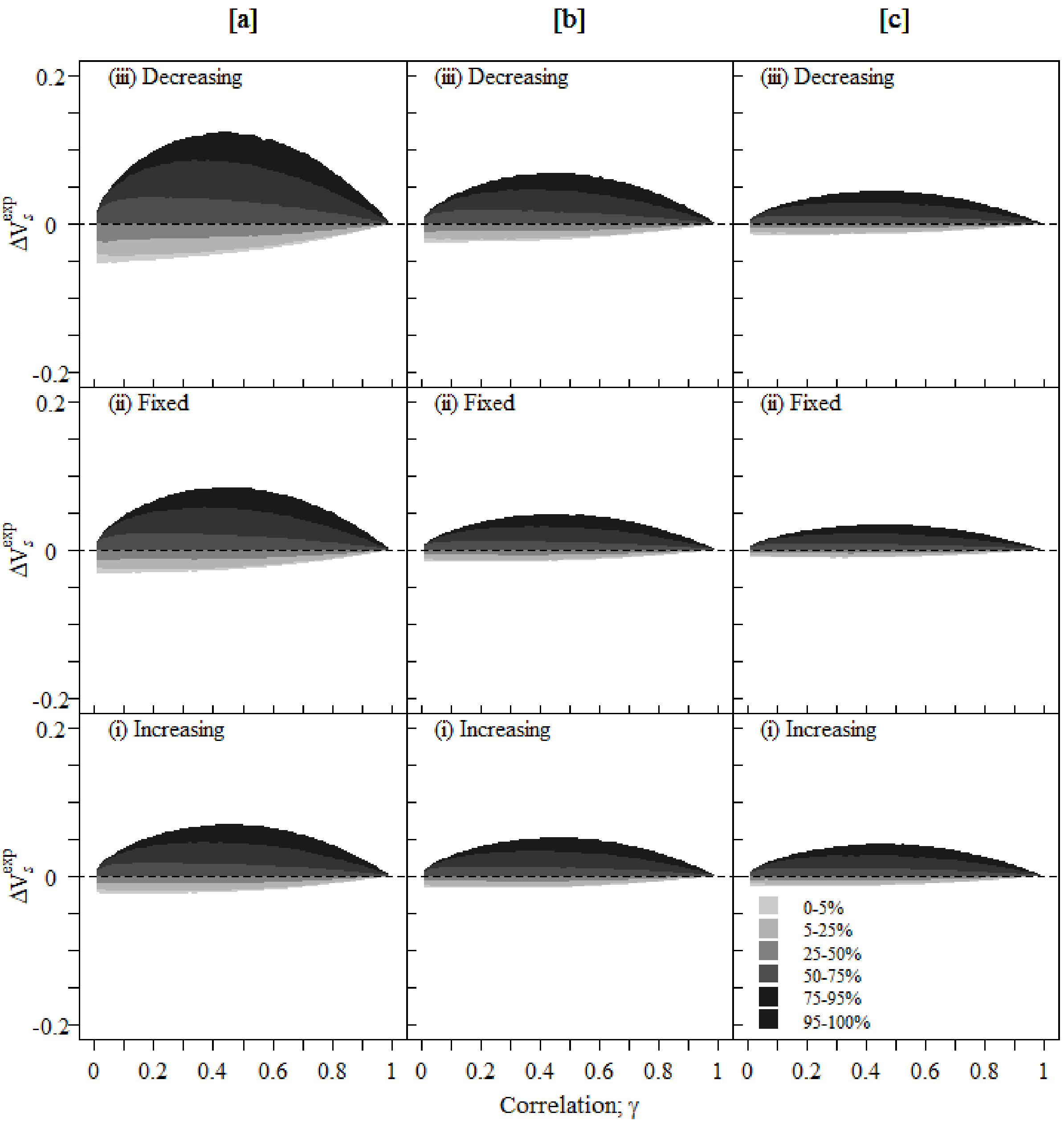

Figure S14: The empirical distribution ($n_{sim} = 10000$) of $\Delta V_s^{\exp}$, the difference from the median value of $V_s^{\exp}$, with varying $1 < d_r < 2$ ($r = 2, 3$) for $s = 4$, with shading showing quantiles 0-5%, 5-25%, 25-50%, 50-75%, 75-95% and 95-100%, for correlations in the range $0 \leq \gamma < 1$ and equal group sizes ($\phi = 0.5$) for [a] early ($\tau_{0_1} = 0.15$), [b] mid ($\tau_{0_2} = 0.30$) and [c] late ($\tau_{0_3} = 0.45$) interim analyses, for the (i) increasing, (ii) fixed and (iii) decreasing rate recruitment models.

2.4 Five outcomes (s=5)

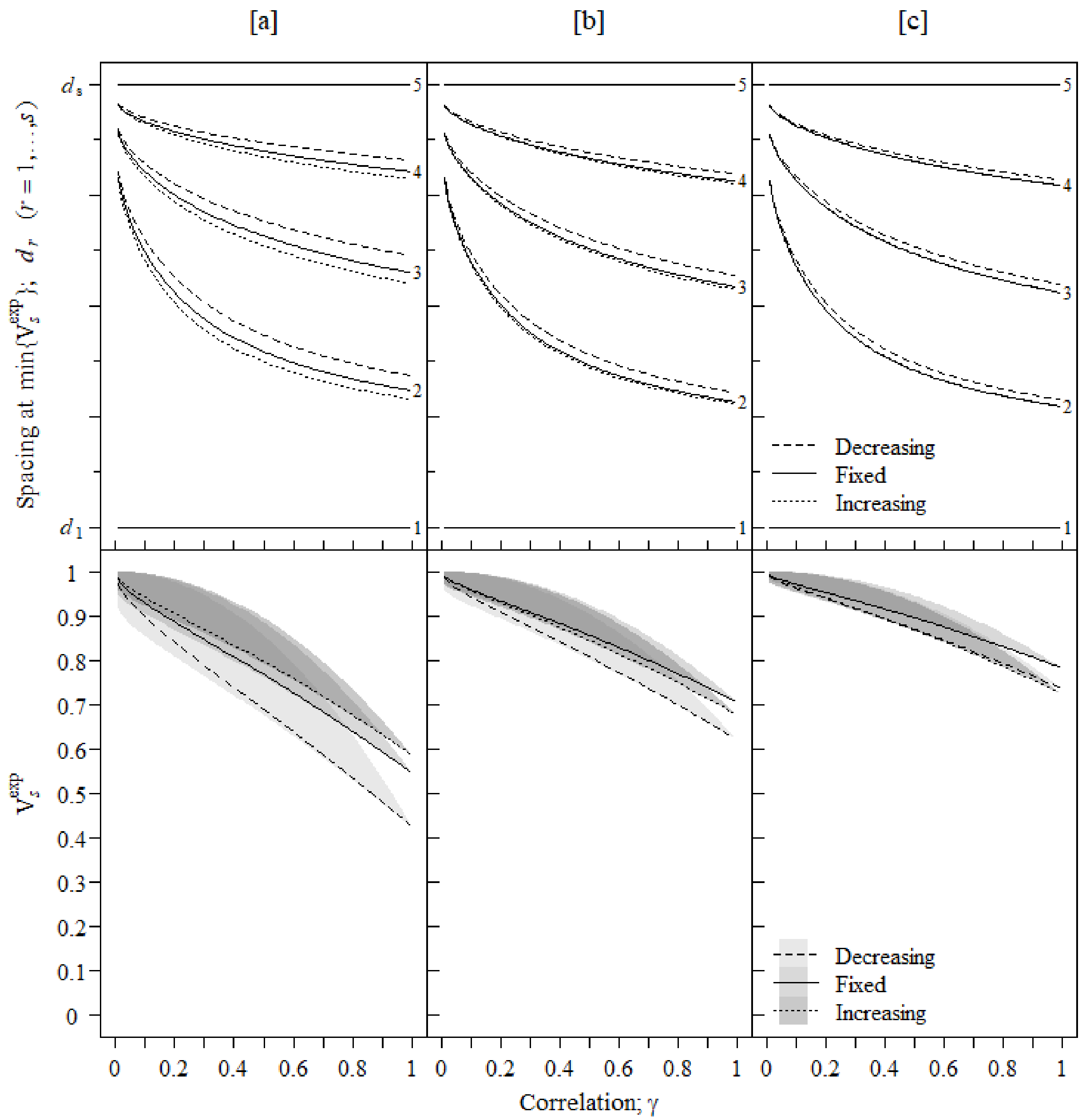

Figure S15: The feasible region of V_s^{exp} for $s = 5$ (shaded areas), bounded above by the maximum and below by the minimum, for correlations in the range $0 \leq \gamma < 1$ and equal group sizes ($\phi = 0.5$) for the decreasing, fixed and increasing rate recruitment models with lines for the setting where the time-points are given by $d_r = 1+(r-1)/(s-1)$ ($r = 1, 2, 3, 4, 5$; i.e. equal spacing) for [a] early ($\tau_{0_1} = 0.15$), [b] mid ($\tau_{0_2} = 0.30$) and [c] late ($\tau_{0_3} = 0.45$) interim analyses.

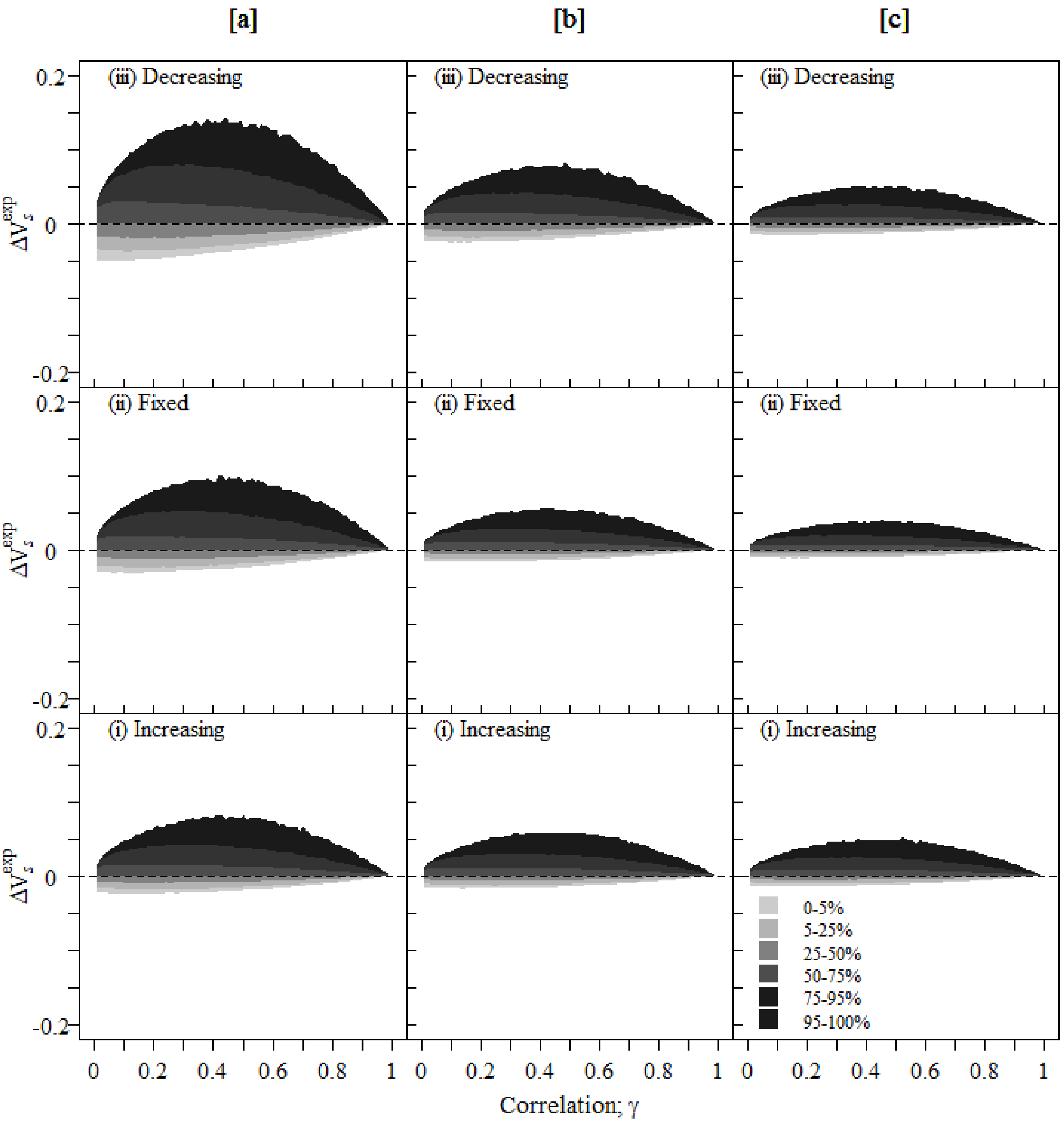

Figure S16: The empirical distribution ($n_{sim} = 10000$) of ΔV_s^{exp}, the difference from the median value of V_s^{exp}, with varying $1 < d_r < 2$ ($r = 2, 3, 4$) for $s = 5$, with shading showing quantiles 0-5%, 5-25%, 25-50%, 50-75%, 75-95% and 95-100%, for correlations in the range $0 \leq \gamma < 1$ and equal group sizes ($\phi = 0.5$) for [a] early ($\tau o_1 = 0.15$), [b] mid ($\tau o_2 = 0.30$) and [c] late ($\tau o_3 = 0.45$) interim analyses, for the (i) increasing, (ii) fixed and (iii) decreasing rate recruitment models.

2.5 Six outcomes (s=6)

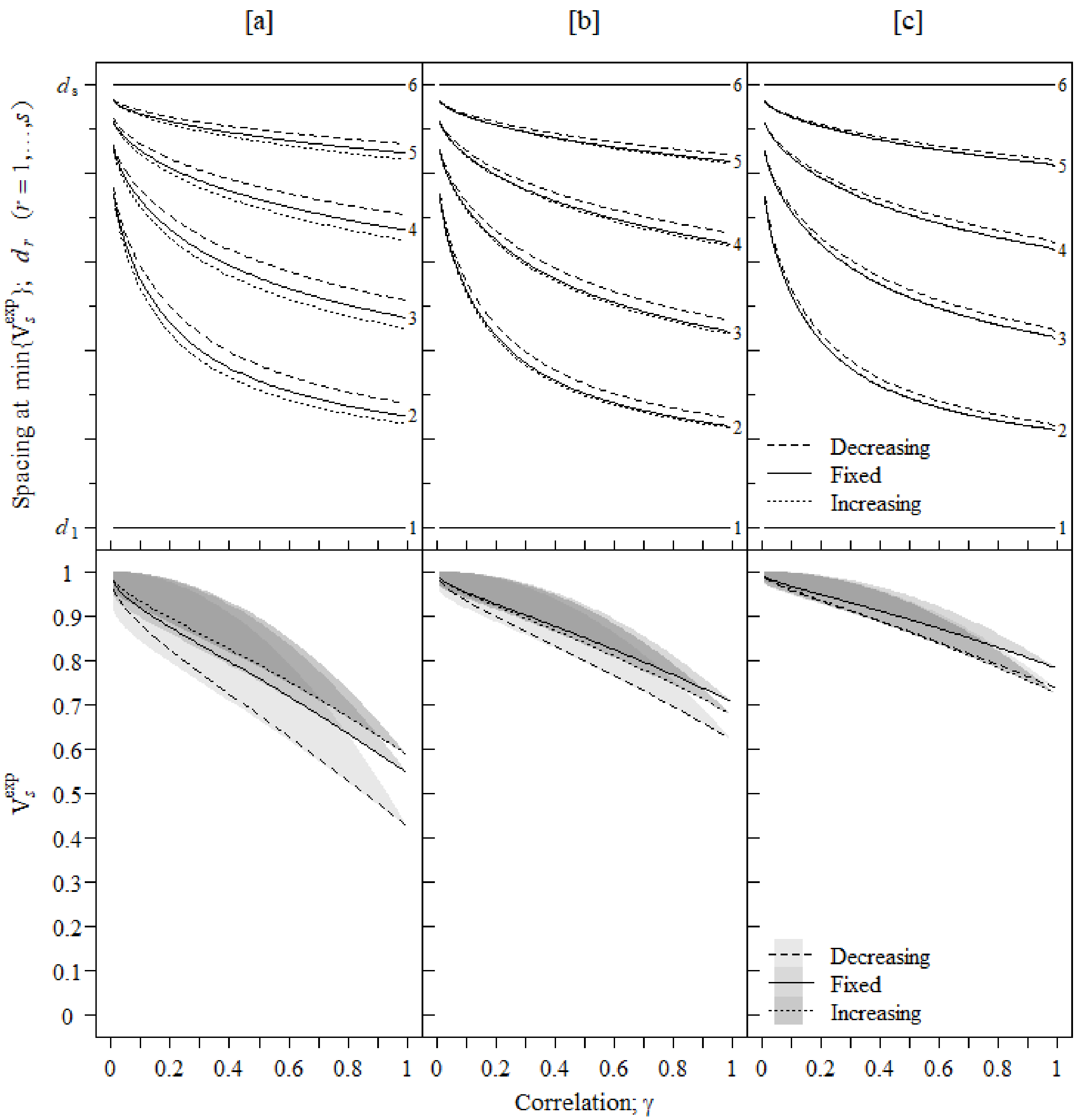

Figure S17: The feasible region of V_s^{exp} for $s = 6$ (shaded areas), bounded above by the maximum and below by the minimum, for correlations in the range $0 \leq \gamma < 1$ and equal group sizes ($\phi = 0.5$) for the decreasing, fixed and increasing rate recruitment models with lines for the setting where the time-points are given by $d_r = 1+(r-1)/(s-1)$ ($r = 1, 2, 3, 4, 5, 6$; i.e. equal spacing) for [a] early ($\tau_{0_1} = 0.15$), [b] mid ($\tau_{0_2} = 0.30$) and [c] late ($\tau_{0_3} = 0.45$) interim analyses.

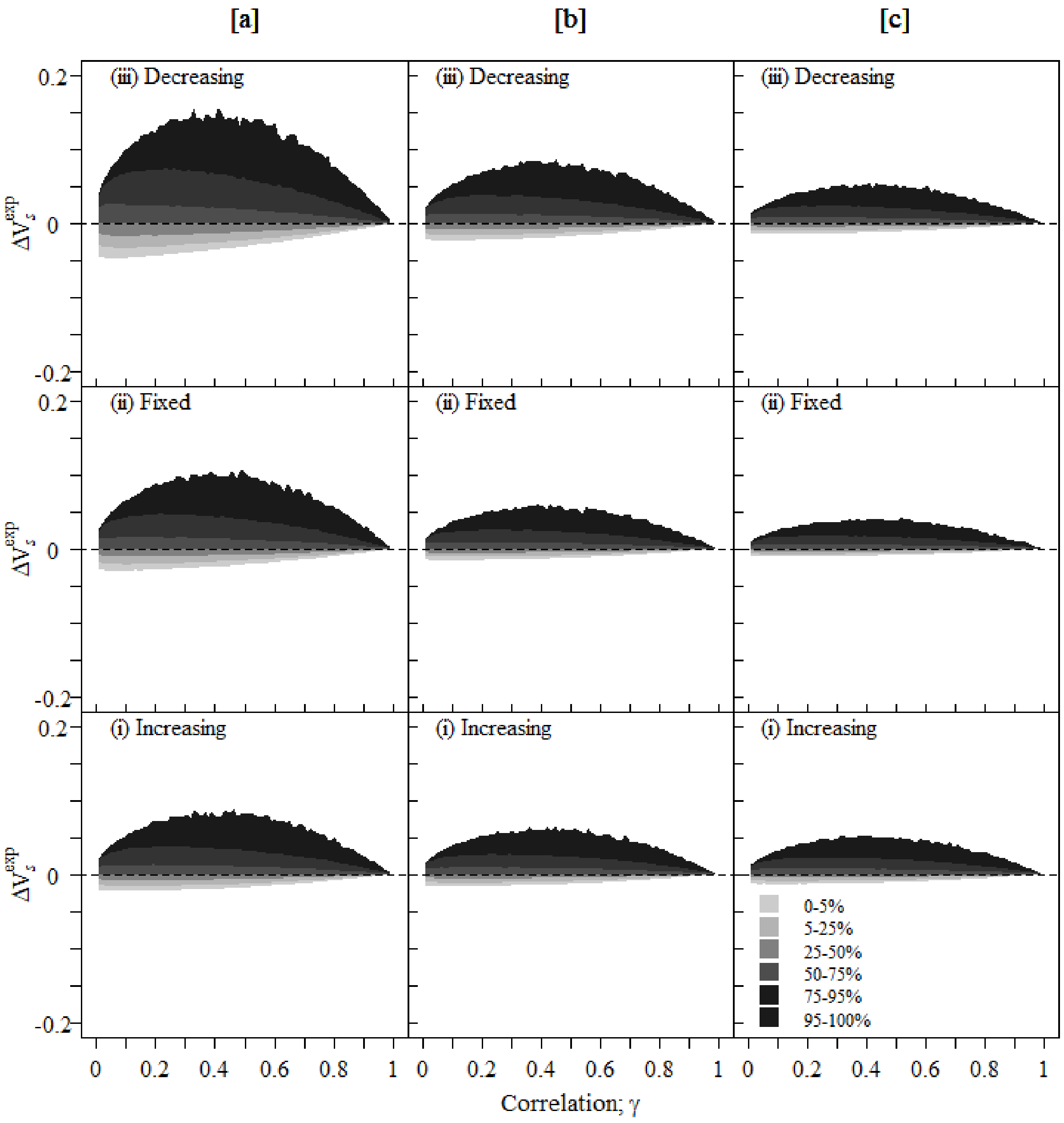

Figure S18: The empirical distribution ($n_{sim} = 10000$) of ΔV_s^{exp}, the difference from the median value of V_s^{exp}, with varying $1 < d_r < 2$ ($r = 2, 3, 4, 5$) for $s = 6$, with shading showing quantiles 0-5%, 5-25%, 25-50%, 50-75%, 75-95% and 95-100%, for correlations in the range $0 \leq \gamma < 1$ and equal group sizes ($\phi = 0.5$) for [a] early ($\tau_{0_1} = 0.15$), [b] mid ($\tau_{0_2} = 0.30$) and [c] late ($\tau_{0_3} = 0.45$) interim analyses, for the (i) increasing, (ii) fixed and (iii) decreasing rate recruitment models.
