# Supplementary material for: Group sequential designs for pragmatic clinical trials with early outcomes: methods and guidance for planning and implementation
Source: BMC Med Res Methodol. 2024 Feb 16;24:42. doi: 10.1186/s12874-024-02174-w (PMC10870612; doi:10.1186/s12874-024-02174-w)
Supplement: Supplementary file 2 — Additional file 1: Appendix A. A.1 Uniform correlation model. A.2 Exponential correlation model. A.3 Partial derivatives of Vexps. A.4 Recruitment and follow-up models. Table A1 Times (t1, t2 and t3) for early τ0(t1) = 0.15, mid τ0(t2) = 0.30 and late τ0(t3) = 0.45 interim analyses, for increasing, fixed and decreasing rate recruitment models. [file 12874_2024_2174_MOESM2_ESM.pdf]

Group sequential designs for pragmatic clinical trials with early outcomes: methods and guidance for planning and implementation

Nick R Parsons[1*], Joydeep Basu[1] and Nigel Stallard[1]

[1]Warwick Clinical Trials Unit (WCTU), Warwick Medical School, University of Warwick, CV4 7AL, Coventry, UK.

*Corresponding author(s). E-mail(s): nick.parsons@warwick.ac.uk;

Appendix A Appendices

A.1 Uniform correlation model

In the most general setting, we assume independence between participants with a multivariate normal distribution for outcomes $(y_{ij1}, \dots, y_{ijs})$, with mean $(\mu_{j1}, \dots, \mu_{js})$ and covariance matrix

$$\Sigma = \mathrm{SRS} = \begin{pmatrix} \sigma_1 & 0 & \dots & 0 \\ 0 & \sigma_2 & \dots & 0 \\ \vdots & \vdots & \ddots & \vdots \\ 0 & 0 & \dots & \sigma_s \end{pmatrix} \begin{pmatrix} 1 & \rho_{12} & \dots & \rho_{1s} \\ \rho_{21} & 1 & \dots & \rho_{2s} \\ \vdots & \vdots & \ddots & \vdots \\ \rho_{s1} & \rho_{s2} & \dots & 1 \end{pmatrix} \begin{pmatrix} \sigma_1 & 0 & \dots & 0 \\ 0 & \sigma_2 & \dots & 0 \\ \vdots & \vdots & \ddots & \vdots \\ 0 & 0 & \dots & \sigma_s \end{pmatrix},$$

where σ_r is the standard deviation of the outcome at occasion r and $\rho_{rr'}$ is the correlation between endpoints at occasions r and $r' = 1, \dots, s$. For the uniform correlation model, R is a $s \times s$ correlation matrix given by

$$\mathrm{R} = \begin{pmatrix} 1 & \alpha & \dots & \alpha \\ \alpha & 1 & \dots & \alpha \\ \vdots & \vdots & \ddots & \vdots \\ \alpha & \alpha & \dots & 1 \end{pmatrix},$$

after setting $\rho_{rr'} = \alpha$ for all occasions $r = 1, \dots, s$ and $r' = 1, \dots, s$ when $r \neq r'$ and $\rho_{rr'} = 1$ when $r = r'$.
The variance of the model parameters $\text{var}(\beta)$ is given by

$$\text{var}(\beta) = \left(\sum_{i=1}^{N} X_i' \Sigma_i^{-1}(\sigma, \alpha) X_i \right)^{-1},$$

where $\Sigma_i(\sigma, \alpha)$ is the covariance matrix of y_i for for participant i, characterised by parameters σ $(\sigma_1, \dots, \sigma_r)$ and α, X_i is a $r \times 2s$ design matrix and β is a $2s \times 1$ vector of unknown model parameters. β can be structured, for convenience, such that $\beta = (\beta_{10}, \beta_{20}, \dots, \beta_{s0}, \beta_1, \beta_2, \dots, \beta_s)$, where β_{s0} estimates the outcome mean in the control arm of the study, and β_r estimates the effect of the treatment arm relative to the control arm at time-point d_r. Therefore, β_s is the effect of the treatment on the study outcome at time-point d_s (the primary endpoint). Noting that $\Sigma^{-1} = \mathrm{S}^{-1}\mathrm{R}^{-1}\mathrm{S}^{-1}$ and

$$\mathrm{R}^{-1} = \frac{1}{(s-1)(\alpha-1)\left(\alpha + \frac{1}{s-1}\right)} \begin{pmatrix} a & b & \dots & b \\ b & a & \dots & b \\ \vdots & \vdots & \ddots & \vdots \\ b & b & \dots & a \end{pmatrix},$$

where $a = -(1 + (s-2)\alpha)$ and $b = \alpha$. Assuming that the number of participants with outcome data are structured such that $N0_1 \geq N0_2 \geq \dots \geq N0_{s-1} \geq N0_s$ and $N1_1 \geq N1_2 \geq \dots \geq N1_{s-1} \geq N1_s$, where $N0_r$ is the number of participants in the control arm and $N1_r$ is the number in the treatment arm at occasion r, and after some algebraic manipulation, we can write

$$\text{var}(\beta_s) = \sigma_s^2 \left[\frac{N0_1 + N1_1}{N0_1 N1_1} + \sum_{m=1}^{s-1} \frac{\det(\mathrm{R}_{m+1})}{\det(\mathrm{R}_m)} \left(\frac{N0_{m+1} + N1_{m+1}}{N0_{m+1} N1_{m+1}} - \frac{N0_m + N1_m}{N0_m N1_m} \right) \right],$$

where $\det(\mathrm{R}_m) = (1-\alpha)^{m-1}(1 + (m-1)\alpha)$ is the determinant of the $m \times m$ uniform correlation matrix R_m.

A.2 Exponential correlation model

Using the same arguments as in section A.1, for the exponential model R_s is a $s \times s$ correlation matrix given by

$$\mathrm{R} = \begin{pmatrix} 1 & \gamma^{d_{12}} & \dots & \gamma^{d_{1s}} \\ \gamma^{d_{21}} & 1 & \dots & \gamma^{d_{2s}} \\ \vdots & \vdots & \ddots & \vdots \\ \gamma^{d_{s1}} & \gamma^{d_{s2}} & \dots & 1 \end{pmatrix},$$

where $d_{rr'} = d_{r'r} = |d_r - d_{r'}|$ is the temporal distance between assessment times d_r and $d_{r'}$, with inverse

$$\mathrm{R}^{-1} = \begin{pmatrix} a_2 & b_2 & 0 & \dots & 0 \\ b_2 & c_3 & b_3 & \ddots & \vdots \\ 0 & b_3 & \ddots & \ddots & 0 \\ \vdots & \ddots & \ddots & c_s & b_s \\ 0 & \dots & 0 & b_s & a_s \end{pmatrix},$$

$$\text{where} \quad a_i = \frac{1}{1-\gamma^{2d_{i(i-1)}}}, \quad b_i = \frac{-\gamma^{2d_{i(i-1)}}}{1-\gamma^{2d_{i(i-1)}}} \quad \text{and}$$

$$c_i = \frac{1-\gamma^{2d_{i(i-2)}}}{(1-\gamma^{2d_{(i-1)(i-2)}})(1-\gamma^{2d_{i(i-1)}})}.$$

Therefore, assuming that the outcome data are structured such that $N0_1 \geq N0_2 \geq \dots \geq N0_{s-1} \geq N0_s$ and $N1_1 \geq N1_2 \geq \dots \geq N1_{s-1} \geq N1_s$, and after some algebraic manipulation, we can write

$$\mathrm{var}(\beta_s) = \sigma_s^2 \Bigg[\frac{(N0_s + N1_s)\{1-\gamma^{2(d_s-d_{s-1})}\}}{N0_s N1_s} + \sum_{m=1}^{s-2} \frac{(N0_{s-m} + N1_{s-m})\{1-\gamma^{2(d_{s-m}-d_{s-m-1})}\}\gamma^{2(d_s-d_{s-m})}}{N0_{s-m} N1_{s-m}} + \frac{(N0_1 + N1_1)\gamma^{2(d_s-d_1)}}{N0_1 N1_1} \Bigg].$$

A.3 Partial derivatives of $\mathrm{V}_s^{\exp}$

For the fixed recruitment rate model (Section 3.1), where $g_r(t, d_r) = (t - d_r)$, the partial derivatives of $\mathrm{V}_s^{\exp}$, see expression (15), with respect to d_{s-m} are

$$\frac{\partial \mathrm{V}_s^{\exp}}{\partial d_{s-m}} = (t-d_s) \Bigg[\frac{2\log(\gamma)\gamma^{2(d_s-d_{s-m})}}{(t-d_{s-m+1})} + \frac{\gamma^{2(d_s-d_{s-m})} - 2(t-d_{s-m})\log(\gamma)\gamma^{2(d_s-d_{s-m})} - \gamma^{2(d_s-d_{s-m-1})}}{(t-d_{s-m})^2} \Bigg],$$

where $m = 1, \dots, s-2$.

For the increasing recruitment rate model (Section 3.2), where $g_r(t, d_r) = (t-d_r)((t-d_r)+1)$, the partial derivatives of $\mathrm{V}_s^{\exp}$ with respect to d_{s-m} are

$$\frac{\partial \mathrm{V}_s^{\exp}}{\partial d_{s-m}} = (t-d_s)((t-d_s)+1) \Bigg[\frac{2\log(\gamma)\gamma^{2(d_s-d_{s-m})}}{(t-d_{s-m+1})((t-d_{s-m+1})+1)} +$$

$$\frac{(2(t-d_{s-m})+1)\gamma^{2(d_s-d_{s-m})}-2(t-d_{s-m})((t-d_{s-m})+1)\log(\gamma)\gamma^{2(d_s-d_{s-m})}}{(t-d_{s-m})^2((t-d_{s-m})+1)^2}-$$

$$\left.\frac{(2(t-d_{s-m})+1)\gamma^{2(d_s-d_{s-m-1})}}{(t-d_{s-m})^2((t-d_{s-m})+1)^2}\right],$$

where $m = 1, \ldots, s-2$.
For the decreasing recruitment rate model (Section 3.3), where $g_r(t, d_r) = (t - d_r)(2\mathrm{T_R} - (t - d_r) + 1)$, the partial derivatives of $\mathrm{V}_s^{\exp}$ with respect to d_{s-m} are

$$\frac{\partial \mathrm{V}_s^{\exp}}{\partial d_{s-m}} = (t-d_s)(2\mathrm{T_R}-(t-d_s)+1)\left[\frac{2\log(\gamma)\gamma^{2(d_s-d_{s-m})}}{(t-d_{s-m+1})(2\mathrm{T_R}-(t-d_{s-m+1})+1)}+\right.$$

$$\frac{(2\mathrm{T_R}-2(t-d_{s-m})+1)\gamma^{2(d_s-d_{s-m})}}{(t-d_{s-m})^2(2\mathrm{T_R}-(t-d_{s-m})+1)^2}-$$

$$\frac{2(t-d_{s-m})(2\mathrm{T_R}-(t-d_{s-m})+1)\log(\gamma)\gamma^{2(d_s-d_{s-m})}}{(t-d_{s-m})^2(2\mathrm{T_R}-(t-d_{s-m})+1)^2}-$$

$$\left.\frac{(2\mathrm{T_R}-2(t-d_{s-m})+1)\gamma^{2(d_s-d_{s-m-1})}}{(t-d_{s-m})^2(2\mathrm{T_R}-(t-d_{s-m})+1)^2}\right],$$

where $m = 1, \ldots, s-2$.

A.4 Recruitment and follow-up models

Setting the recruitment period $\mathrm{T_R}$ to be a multiple m of the primary (final) outcome time d_s, $\mathrm{T_R} = md_s$, where $m > 1$. Typically in pragmatic clinical trials the length of recruitment might be two ($m = 2$), three ($m = 3$) or four ($m = 4$) times the final outcome time-point; e.g. for a trial with a 12 month outcome then recruitment might take 24, 36 or 48 months to complete.
If t_f is the time period, after the study primary outcome, when an interim analysis at time t occurs (i.e. $t_f = t - d_s$), then from Section 3.1, for the fixed rate recruitment model

$$N_s(t, d_s) = \frac{N(t-d_s)}{\mathrm{T_R}} = \frac{Nt_f}{md_s} \qquad \text{and} \qquad \frac{N_s(t, d_s)}{N} = \frac{t_f}{md_s}.$$

At an interim look at *information fraction* $\tau 0$ (i.e. $\tau 0 = N_s(t, d_s)/N$) we might typically require some proportion $\tau 0$, where $0 < \tau 0 \leq 1$, of the study participants to have primary outcome data available for analysis and for the fixed rate recruitment model this will be at time $t_f = \tau 0 m d_s$ after the study primary outcome time at d_s. The full study length will be given by $d_s + t_f = d_s + \tau 0 m d_s$ for $\tau 0 = 1$ which is $d_s + \mathrm{T_R}$, when follow-up on the final participant recruited at $\mathrm{T_R}$ is complete.
If t_i is the time period, after the study primary outcome, when an interim analysis at time t occurs (i.e. $t_i = t - d_s$), then from Section 3.2, for the increasing rate recruitment model

$$N_s(t,d_s) = \frac{N(t-d_s)((t-d_s)+1)}{\mathrm{T_R}(\mathrm{T_R}+1)} = \frac{Nt_i(t_i+1)}{md_s(md_s+1)}$$
$$\text{and} \qquad \frac{N_s(t,d_s)}{N} = \frac{t_i(t_i+1)}{md_s(md_s+1)}.$$

As we require the interim analysis for the increasing rate model to be at the same value of $\tau 0$ as used for the fixed rate model we can set $\tau 0 = t_f/md_s$ for $N_s(t,d_s)/N$ in the above to get the expression $t_i^2 + t_i - t_f(md_s+1) = 0$ which has solution

$$t_i = \frac{-1+\sqrt{1+4t_f(md_s+1)}}{2}.$$

As a check, we note that when $\tau 0 = 1$ (and $t_f = md_s$) the study has completed follow-up and then from the above expression $t_i = t_f$, as we would expect.

If t_d is the time period, after the study primary outcome, when an interim analysis at time t occurs (i.e. $t_d = t - d_s$), then from Section 3.3, for the decreasing rate recruitment model

$$N_s(t,d_s) = \frac{N(t-d_s)(2\mathrm{T_R}-(t-d_s)+1)}{\mathrm{T_R}(\mathrm{T_R}+1)} = \frac{Nt_d(2md_s-t_d+1)}{md_s(md_s+1)}$$
$$\text{and} \qquad \frac{N_s(t,d_s)}{N} = \frac{t_d(2md_s-t_d+1)}{md_s(md_s+1)}.$$

As we require the interim analysis for the increasing rate model to be at the same value of $\tau 0$ as used for the fixed rate model we can set $\tau 0 = t_f/md_s$ for $N_s(t,d_s)/N$ in the above to get the expression $-t_d^2 + t_d(2md_s+1) - t_f(md_s+1) = 0$ which has solution

$$t_d = \frac{-(2md_s+1)+\sqrt{(2md_s+1)^2-4t_f(md_s+1)}}{-2}.$$

As a check, we note that when $\tau 0 = 1$ (and $t_f = md_s$) the study has completed follow-up and then from the above expression $t_d = t_i = t_f$, as we would expect.
In the setting described in the manuscript, we choose $m = 4$, $d_s = 2$ and three equally spaced interim analyses at $\tau 0(t_1) = 0.15$, $\tau 0(t_2) = 0.30$ and $\tau 0(t_3) = 0.45$. Therefore, for the fixed model at the first interim analysis, ($\tau 0(t_1) = 0.15$), $t_{f1} = \tau 0_1 md_s = 0.15 \times 4 \times 2 = 1.2$ and thus the first interim analysis is at time $d_1 = d_s + t_{f1} = 2+1.2 = 3.2$. The second ($\tau 0(t_2) = 0.30$) at $d_2 = d_s + t_{f2} = 2+2.4 = 4.4$ and the third ($\tau 0(t_3) = 0.45$) at $d_3 = d_s + t_{f3} = 2 + 3.6 = 5.6$. For the increasing and decreasing rate models the above formulation yields the values in the Table A1.

Table A1 Times (t_1, t_2 and t_3) for early $\tau 0(t_1) = 0.15$, mid $\tau 0(t_2) = 0.30$ and late $\tau 0(t_3) = 0.45$ interim analyses, for increasing, fixed and decreasing rate recruitment models.

Rate	Early<br>t_1	Mid<br>t_2	Late<br>t_3
Increasing	4.82	6.17	7.21
Fixed	3.20	4.40	5.60
Decreasing	2.66	3.38	4.19
